# Controlled-release fertilizer programs differentially affect leaf nutrition and nutrient factor productivity in mature HLB-affected ‘Valencia’ sweet orange trees in Florida

**DOI:** 10.3389/fpls.2026.1932128

**Published:** 2026-09-28

**Authors:** Jonas Pereira de Souza Junior, Jude W. Grosser, Davie Mayeso Kadyampakeni

**Affiliations:** 1 Citrus Research and Education Center, Soil, Water and Ecosystem Sciences Department, University of Florida, Lake Alfred, FL, United States; 2 Citrus Research and Education Center, Horticultural Sciences Department, University of Florida, Lake Alfred, FL, United States

**Keywords:** citrus nutrition, *Citrus sinensis*, fertilizer management, nutrient-use efficiency, partial factor production

## Abstract

Huanglongbing (HLB) compromises citrus root function, nutrient uptake, and productivity, making fertilizer management critical for sustaining tree performance in affected groves. This study evaluated conventional and controlled-release fertilizer (CRF) programs in mature HLB-affected ‘Valencia’ sweet orange trees grown on sandy soil in Florida. Treatments consisted of a conventional standard fertilizer program (Standard) and two CRF programs, CRF1 and CRF2. Leaf nutrient concentrations, fruit yield, juice quality, partial factor productivity (PFP), and multivariate response profiles were assessed. CRF1 increased selected leaf macronutrient concentrations, particularly magnesium (Mg) and sulfur (S), whereas micronutrient responses were limited mainly to manganese (Mn). Fruit yield did not differ among treatments in 2024 or 2025; however, CRF2 reduced yield in 2026 compared with Standard and CRF1. Juice quality attributes were not significantly affected by fertilization program. In contrast, PFP was more responsive to nutrient management, with CRF2 showing the highest PFP for potassium (PFP-K_2_O) in both 2025 and 2026. Hierarchical clustering separated CRF1 from Standard and CRF2, indicating a distinct integrated nutritional and productive response profile. Principal component analysis showed that treatment discrimination was mainly driven by PFP indices, especially PFP for nitrogen (PFP-N) and PFP for phosphorus (PFP-P_2_O_5_), together with fruit yield in 2026. Ridge regression indicated that yield was positively associated mainly with leaf S and nitrogen (N) under CRF2 and leaf phosphorus (P) under CRF1, whereas leaf calcium (Ca) under CRF2 showed the strongest negative association with yield. Overall, controlled-release fertilizer programs were not agronomically interchangeable: CRF1 maintained yield and improved nutritional status, whereas CRF2 increased apparent PFP-K_2_O but did not sustain yield stability.

## Graphical Abstract

**Figure:**
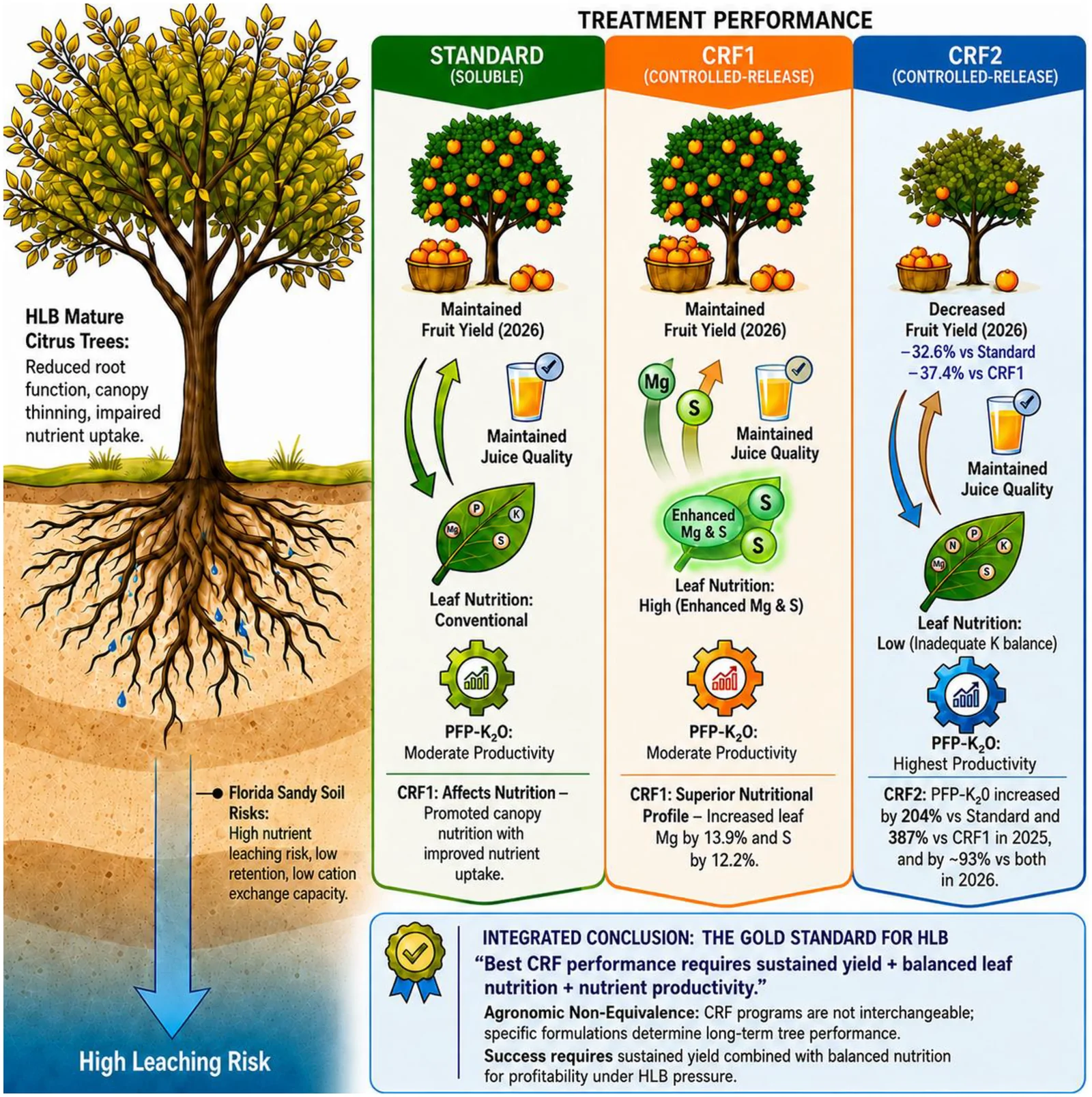

## 1 Introduction

Citrus production in Florida has been devastated by huanglongbing (HLB), a destructive systemic disease caused by *Candidatus* Liberibacter asiaticus (Graham et al., 2024). Trees affected by HLB typically exhibit canopy thinning, root decline, nutritional imbalances, yield reduction, and diminished fruit marketability due to disrupted phloem transport, compromised root health, limited water and nutrient uptake, and reduced tree productivity and fruit quality (Silva et al., 2021; Cifuentes-Arenas et al., 2022). In the absence of a widely adopted cure under field conditions, integrated management approaches focused on preserving tree health and sustaining productivity are critical for maintaining citrus production in HLB-affected groves.

Nutrient management represents a key strategy for supporting the performance of HLB-affected citrus trees, particularly because disease-induced root decline can constrain nutrient uptake even when nutrients are present in the soil solution (Uthman et al., 2022; Atta et al., 2023). This challenge is especially relevant in Florida citrus production systems, where sandy soils commonly exhibit low organic matter content, low cation exchange capacity, limited nutrient retention, and high vulnerability to nutrient leaching (Atta et al., 2022). Under such conditions, conventional soluble fertilizers may fail to adequately synchronize nutrient supply with tree demand, particularly as rainfall, irrigation, root health, and nutrient mobility interact dynamically in the root zone (Atta et al., 2020b; Luo et al., 2023). Consequently, fertilizer source, application timing, nutrient balance, and release pattern are important considerations for improving nutrient availability and uptake efficiency in HLB-affected citrus groves.

Controlled-release fertilizers (CRFs) have been proposed as either an alternative or a complementary approach to conventional dry soluble fertilizer applications because they release nutrients gradually over time, thereby potentially reducing nutrient losses and improving the temporal synchronization between nutrient supply and plant demand (Govil et al., 2024). In citrus production systems, CRFs have been investigated as a means of improving nutrient-use efficiency, reducing nitrate leaching, and sustaining tree nutritional status under sandy soil conditions (Zekri and Koo, 1992; El Bey et al., 2021; Ma et al., 2022). Nevertheless, CRF-based fertilization programs are not necessarily agronomically interchangeable, because their performance may vary according to fertilizer formulation, nutrient ratios, total nutrient input, release duration, and application frequency. Previous studies have demonstrated that differences in CRF composition, release period, application rate, and the proportion of controlled-release relative to conventional fertilizer can markedly affect nutrient availability, nutrient-use efficiency, and crop productivity (Hu et al., 2023; Govil et al., 2024; Yin et al., 2026). Consequently, direct comparisons between distinct CRF-based and conventional fertilization programs are needed to determine whether commercially relevant fertilizer strategies differing in formulation, nutrient inputs, release characteristics, and application schedules produce distinct nutritional and productive responses under HLB-affected field conditions. Accordingly, the present study evaluates complete fertilization programs rather than the isolated effect of controlled-release technology, and treatment responses should therefore be interpreted as the integrated outcome of fertilizer formulation, nutrient rate, release characteristics, and application schedule.

This distinction is particularly relevant in mature HLB-affected citrus groves, where compromised root systems and variable nutrient acquisition can reduce the predictability of fertilizer responses (Atta et al., 2022). Although enhanced nutritional programs have been widely considered as part of HLB management (Dong et al., 2021; Kwakye and Kadyampakeni, 2022), field responses remain inconsistent. Previous studies have demonstrated that nutrient management can affect root growth, leaf nutrient status, canopy development, yield, and fruit quality in HLB-affected citrus trees solution (Atta et al., 2022, 2023; Uthman et al., 2022). Moreover, recent evidence from pomelo production systems showed that reducing phosphorus (P) fertilizer inputs can maintain fruit yield while improving selected fruit-quality attributes, highlighting that greater nutrient inputs do not necessarily translate into superior agronomic performance (Luo et al., 2025). However, such responses are often site-specific and strongly influenced by nutrient source, application rate, application method, soil nutrient availability, and environmental conditions.

Despite the potential benefits of CRFs, it remains unclear whether different controlled-release fertilizer programs produce similar or contrasting responses compared with conventional fertilization in mature HLB-affected citrus trees grown on sandy soil. This knowledge gap is agronomically important because CRF performance depends on the use of controlled-release technology and on fertilizer formulation, nutrient ratios, release duration, total nutrient input, and application timing. Therefore, direct comparisons among CRF programs are necessary to determine whether these fertilizer strategies are functionally equivalent or whether specific formulations generate distinct nutritional and productive outcomes under HLB pressure.

We hypothesized that the two controlled-release fertilizer programs, CRF1 and CRF2, would not be agronomically interchangeable and would produce distinct responses compared with the conventional standard program by differentially affecting leaf nutrient concentrations, fruit yield, juice quality, and nutrient productivity. Therefore, the objective of this study was to compare the effects of two controlled-release fertilizer programs and a conventional standard fertilization program on leaf nutrient concentrations, fruit yield, juice quality, partial factor productivity, and multivariate response profiles in mature HLB-affected ‘Valencia’ sweet orange trees grown on sandy soil in Florida.

## 2 Materials and methods

### 2.1 Location and characteristics of the study area

The experiment was conducted from January 2024 to April 2026, encompassing winter, spring, summer, and autumn growing conditions, in a mature citrus grove located in Lake Alfred, Florida, USA (28.112586° N, 81.712599° W), planted with ‘Valencia’ sweet orange (*Citrus sinensis* L.) trees grafted onto Swingle citrumelo (*Citrus paradisi* Macf. × *Poncirus trifoliata* (L.) Raf.) rootstocks. The trees were planted in February 2013; therefore, the trees were 11 years old at the onset of the study in 2024. The soil at the experimental site was classified as Candler fine sand, with a particle-size distribution of 944.6 g kg^-1^ sand, 34.3 g kg^-1^ clay, and 21.1 g kg^-1^ silt, and taxonomically identified as hyperthermic, uncoated Lamellic Quartzipsamments (Soil-Survey-Staff, 2014).

The study area was embedded within a larger commercial grove, where adjacent trees were managed under conventional practices, including fertilization, liming, agrichemical applications, and mechanical weed control. Seasonal mean temperature, relative humidity, cumulative rainfall, and wind speed were obtained from a local weather station located approximately 0.5 km from the experimental site (**Figure 1**). During the experimental period, the site experienced seasonal chilling conditions from January to March 2024, December 2024 to February 2025, and November 2025 to March 2026 (including rare severe freezes from January 26 to February 5). The study area was also affected by Hurricane Milton in October 2024.

**Figure 1:**
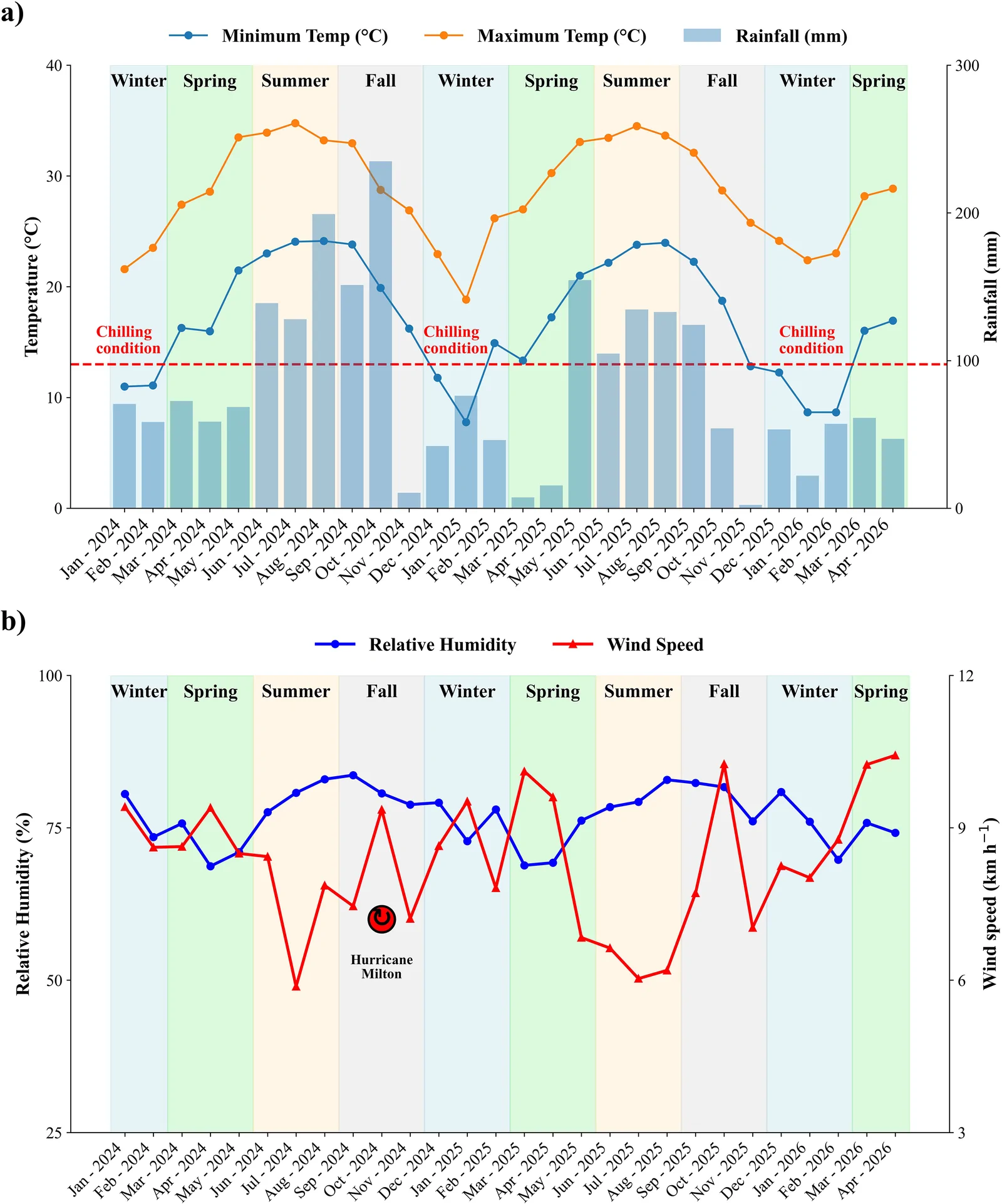
Monthly meteorological conditions at the experimental site in Lake Alfred, Florida, from January 2024 to April 2026. **(A)** Monthly minimum and maximum air temperature (°C) and cumulative rainfall (mm). **(B)** Mean monthly relative humidity (%) and wind speed (km h^⁻¹^). Shaded areas indicate seasonal periods. The dashed horizontal line in panel A indicates the temperature threshold associated with chilling conditions. The occurrence of Hurricane Milton in October 2024 is indicated in panel B. Meteorological data were obtained from a weather station located approximately 0.5 km from the experimental site.

Trees were spaced 4.57 m within rows and 3.05 m between rows, corresponding to a planting density of 717 trees ha^-1^. The experimental area was divided into 24 plots, each comprising 33 trees. Adjacent plots were separated by at least three buffer trees to minimize confounding border effects. Within each plot, ten central trees were selected for detailed assessments, including soil and leaf sampling, vegetative growth measurements, fruit yield determination, and juice quality analysis.

Prior to treatment establishment, soil samples were collected from the 0–20 and 20–40 cm depth intervals and submitted for chemical characterization. Soil fertility was assessed following the standard soil testing programs described by Mylavarapu et al. (2014). Initial soil chemical properties for the 0–20 and 20–40 cm depths, respectively, were: pH in water, 6.06 and 5.48; P, 195.0 and 212.7 mg kg^-1^; K, 33.4 and 18.8 mg kg^-1^; Ca, 778.2 and 388.2 mg kg^-1^; Mg, 64.5 and 30.7 mg kg^-1^; S, 40.5 and 27.1 mg kg^-1^; organic matter, 6.5 and 3.8 g kg^-1^; and cation exchange capacity, 5.68 and 3.59 cmolc kg^-1^.

Trees also exhibited typical symptoms of huanglongbing (HLB). Infection by *Candidatus Liberibacter asiaticus* was confirmed by TaqMan quantitative polymerase chain reaction (qPCR) targeting the bacterial 16S rRNA gene, following the method described by Li et al. (2006). The assay used the CLas-specific forward primer HLBas (5′-TCGAGCGCGTATGCAATACG-3′), reverse primer HLBr (5′-GCGTTATCCCGTAGAAAAAGGTAG-3′), and probe HLBp (5′-FAM-AGACGGGTGAGTAACGCG-BHQ-3′). Positive CLas DNA and no-template controls were included in each assay, and the citrus mitochondrial cytochrome oxidase gene was used as an endogenous amplification control. The analyzed sample produced a Ct value of 28.94, and was therefore classified as CLas-positive. In addition, a visual assessment of canopy decline was performed at the plot level using field photographs obtained from all experimental units. Canopy condition was rated using an ordinal scale from 0 to 5, where 0 = no visible decline, 1 = slight decline, 2 = mild-to-moderate decline, 3 = moderate decline, 4 = severe decline, and 5 = very severe decline, based on canopy thinning and visible branch dieback. This assessment was used as a supplementary indicator of the general canopy condition of HLB-affected trees (**Figure 2**).

**Figure 2:**
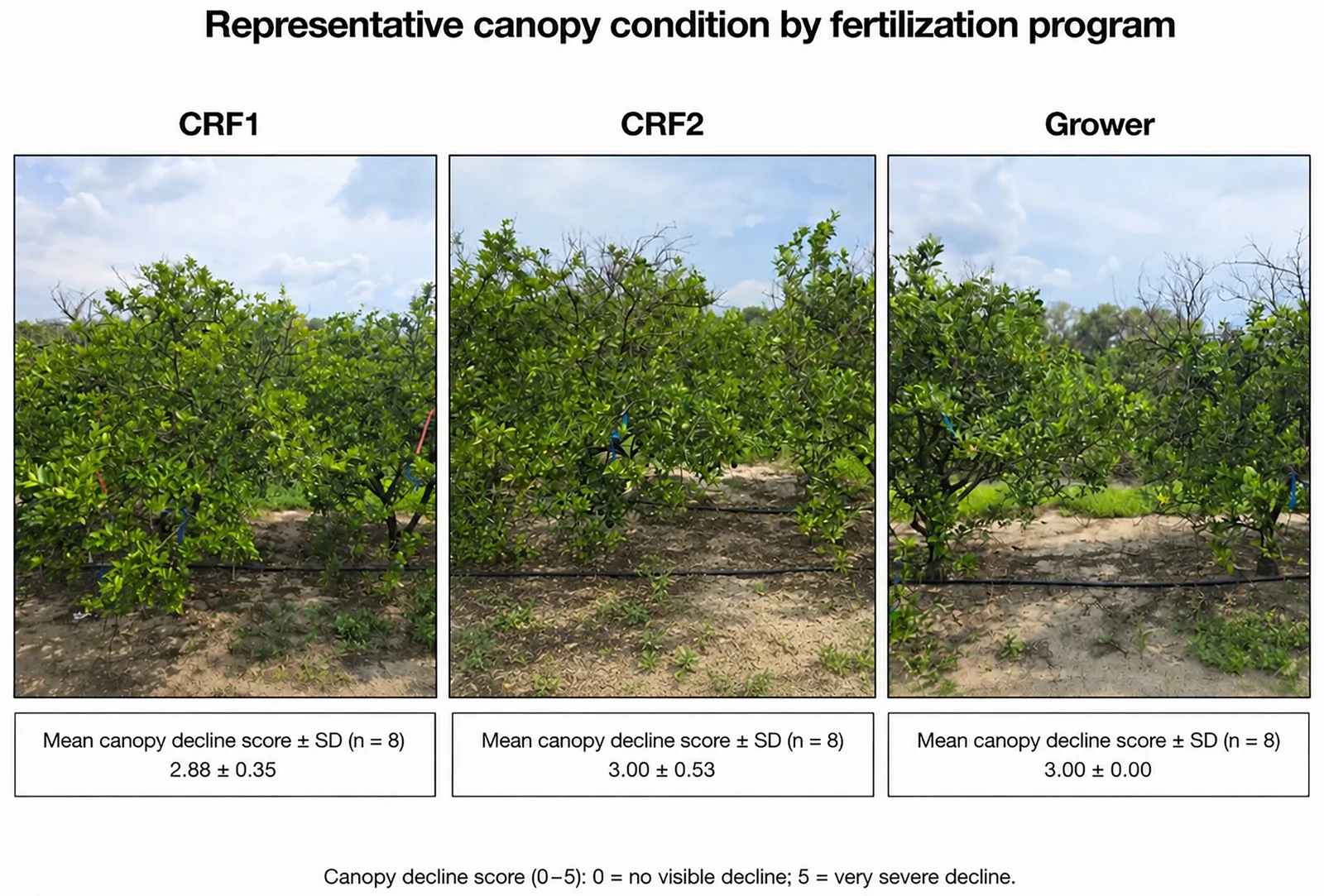
Representative canopy condition of mature HLB-affected ‘Valencia’ sweet orange trees under the three fertilization programs: controlled-release fertilizer program 1 (CRF1), controlled-release fertilizer program 2 (CRF2), and the conventional Standard program. Mean canopy decline scores were 2.88 ± 0.35 for CRF1, 3.00 ± 0.53 for CRF2, and 3.00 ± 0.00 for Standard. Canopy decline was visually assessed using an ordinal scale from 0 to 5, where 0 = no visible decline, 1 = slight decline, 2 = mild-to-moderate decline, 3 = moderate decline, 4 = severe decline, and 5 = very severe decline, based on canopy thinning and visible branch dieback.

### 2.2 Study design and experimental plot

The experiment was arranged in a randomized complete block design with three fertilization treatments and eight replicates, resulting in 24 experimental plots. The treatments included a conventional fertilization program, hereafter referred to as Standard, and two controlled-release fertilizer programs, referred to as CRF1 and CRF2. Fertilizer rates originally applied on a per-tree basis were converted to a hectare basis using the planting density of 717 trees ha^-1^. Fertilizer composition was described according to the guaranteed analysis of each product, and nutrient concentrations are expressed as percentage of product mass.

The treatments were evaluated as complete fertilization programs rather than as isolated fertilizer sources. Accordingly, the programs differed simultaneously in fertilizer technology, nutrient formulation, application timing, application frequency, nutrient ratios, and total nutrient inputs. This approach was adopted to compare commercially relevant fertilization strategies under field conditions in mature HLB-affected citrus trees, where fertilizer performance reflects the combined effects of nutrient source, formulation, rate, release pattern, and application schedule. Because these factors were not independently controlled, the effects of controlled-release technology cannot be separated from those associated with nutrient composition and management strategy. Therefore, treatment responses should be interpreted as the integrated effects of the complete fertilization program rather than as isolated effects of controlled-release fertilizer technology.

The Standard treatment consisted of a 10–2–13 fertilizer containing 10% nitrogen (N), 2% available phosphate (P_2_O_5_), 13% soluble potash (K_2_O), 5% calcium (Ca), 2.5% magnesium (Mg), 12.76% sulfur (S), 0.03% boron (B), 0.272% iron (Fe), 0.71% zinc (Zn), and 0.71% manganese (Mn). This fertilizer was applied at 559.3 kg ha^-1^ per application. During the experimental period, the Standard treatment received one application in October 2024, three applications in April, June, and October 2025, and one application in March 2026.

The CRF1 treatment consisted of a 5–1–7 controlled-release fertilizer containing 5% total N, 1% available phosphate (P_2_O_5_), 7% soluble potash (K_2_O), and no more than 2% chlorine (Cl). In addition, it contained 3.204% Ca, 4.029% Mg, 10.355% S, 0.331% B, 0.280% copper (Cu), 4.198% Fe, 3.838% Mn, 0.023% molybdenum (Mo), and 2.009% Zn. According to the guaranteed analysis, part of the N, phosphate, potash, Mg, Fe, Mn, and Zn fractions was supplied in slow- or controlled-release forms, while selected micronutrients were also present in chelated forms. The CRF1 fertilizer was applied at 1,681 kg ha^-1^ per application. During the experimental period, CRF1 received one application in October 2024, one application in September/October 2025, and one application in March/April 2026.

The CRF2 treatment consisted of a split-fertilization program with two fertilizer formulations and an application schedule distinct from that of CRF1. Therefore, differences between CRF1 and CRF2 should be interpreted as the integrated effects of fertilizer composition, nutrient inputs, release characteristics, and application timing rather than as differences in controlled-release technology alone. The 10–2–3 fertilizer contained 10% N, 2% P_2_O_5_, and 3% K_2_O, whereas the 5–1–5 controlled-release fertilizer was a 180-day release product containing 5% N, 1% P_2_O_5_, 5% K_2_O, 3% Ca, 4% Mg, 12% S, 0.15% B, 0.10% Cu, 4% Fe, 3% Mn, 0.002% Mo, and 2% Zn. For the 5–1–5 fertilizer, N was derived from polymer-coated ammonium nitrate and potassium nitrate; P was derived from polymer-coated ammoniated phosphate; and K was derived from polymer-coated sulfate of potash, potassium nitrate, and K-Mag. Micronutrients were supplied in polymer-coated and/or chelated forms. During the experimental period, CRF2 received one 5–1–5 application in October 2024 at 559.4 kg ha^-1^; three applications in 2025, consisting of 5–1–5 in April at 559.4 kg ha^-1^, 10–2–3 in June at 673.2 kg ha^-1^, and 5–1–5 in October at 559.4 kg ha^-1^; and one 10–2–3 application in March 2026 at 673.2 kg ha^-1^. Because CRF2 was a split-fertilization program, the nutrient composition applied in each year depended on the formulation used. In 2024, CRF2 received only the 5–1–5 controlled-release formulation, whereas in 2025 it received two applications of 5–1–5 and one application of 10–2–3. In 2026, CRF2 received only the 10–2–3 formulation. Therefore, annual inputs of Ca, Mg, S, and micronutrients under CRF2 originated from the 5–1–5 applications, whereas the 2026 application contributed only the nutrients listed in the guaranteed analysis available for the 10–2–3 formulation.

Annual and cumulative nutrient inputs supplied by each fertilization program over the experimental period were calculated from the guaranteed analyses and the total amount of fertilizer applied (**Table 1**).

**Table 1:** Annual and cumulative nutrient inputs supplied by the standard, CRF1, and CRF2 fertilization programs during the experimental period.

Program	Period	N	P_2_O_5_	K_2_O	Ca	Mg	S	B	Cu	Fe	Mn	Mo	Zn
		-–- kg ha^-1^ -–-											
Standard	2024	55.93	11.19	72.71	27.96	13.98	71.37	0.17	n.l.	1.52	3.97	n.l.	3.97
Standard	2025	167.79	33.56	218.13	83.89	41.95	214.10	0.50	n.l.	4.56	11.91	n.l.	11.91
Standard	2026	55.93	11.19	72.71	27.96	13.98	71.37	0.17	n.l.	1.52	3.97	n.l.	3.97
Standard	**Cumulative**	**279.65**	**55.93**	**363.54**	**139.82**	**69.91**	**356.83**	**0.84**	**n.l.**	**7.61**	**19.86**	**n.l.**	**19.86**
CRF1	2024	84.05	16.81	117.67	53.86	67.73	174.07	5.56	4.71	70.57	64.52	0.39	33.77
CRF1	2025	84.05	16.81	117.67	53.86	67.73	174.07	5.56	4.71	70.57	64.52	0.39	33.77
CRF1	2026	84.05	16.81	117.67	53.86	67.73	174.07	5.56	4.71	70.57	64.52	0.39	33.77
CRF1	**Cumulative**	**252.15**	**50.43**	**353.01**	**161.58**	**203.18**	**522.20**	**16.69**	**14.12**	**211.71**	**193.55**	**1.16**	**101.31**
CRF2	2024	27.97	5.59	27.97	16.78	22.38	67.13	0.84	0.56	22.38	16.78	0.01	11.19
CRF2	2025	123.26	24.65	76.14	33.56	44.75	134.26	1.68	1.12	44.75	33.56	0.02	22.38
CRF2	2026	67.32	13.46	20.20	n.l	n.l	n.l	n.l	n.l	n.l	n.l	n.l	n.l
CRF2	**Cumulative**	**218.55**	**43.71**	**124.30**	**50.35**	**67.13**	**201.38**	**2.52**	**1.68**	**67.13**	**50.35**	**0.03**	**33.56**

n.l., nutrient not listed in the guaranteed analysis of the fertilizer formulation. The annual and cumulative nutrient inputs further demonstrate that the treatments represented distinct fertilization programs differing in fertilizer-release characteristics, nutrient composition, application schedule, and total nutrient inputs.

The bold value is the cumulative for the three years season.

### 2.3 Soil and leaf sampling for determining nutritional concentration

Soil and leaf samples were collected annually in 2024, 2025, and 2026 near harvest. Soil subsamples were collected from both sides of the central trees within each plot at two depths: 0–20 and 20–40 cm. Soil samples were oven-dried at 100 °C for 24 h, sieved, and analyzed for pH, extractable nutrients, organic matter, and cation exchange capacity following standard soil testing procedures.

In the laboratory, a 5 g subsample of each dried and sieved soil sample was measured using a Standard North Central Region (NCR) 5 g scoop and transferred to test tubes containing 25 mL of 0.5 M acetic acid. Samples were mixed for 2 min, allowed to stand for 24 h, and subsequently shaken for 2 h on a shaker table at 200 rpm with a 2.54 cm throw. After extraction, samples were filtered through Whatman No. 1 filter paper, and the filtrate was transferred to 15 × 100 mm disposable polypropylene test tubes. Extractable P, K, Ca, Mg, and S were quantified by inductively coupled plasma–atomic emission spectroscopy (ICP-AES). The same analytical protocol was used for all treatments to ensure comparability among soil samples.

A total of five fully expanded leaves were collected from both sides of each of the five central trees per plot and combined to form one composite leaf sample per plot. Leaf samples were washed sequentially with water, detergent solution (0.1%, w/w), HCl solution (0.3%), and deionized water to remove surface contaminants. Thereafter, samples were oven-dried at 65 °C until constant dry mass, ground, and chemically digested for nutrient determination. Leaf N, P, K, Ca, Mg, S, B, Zn, Mn and Cu concentrations were determined using standard plant tissue analysis procedures.

### 2.4 Plant development and biometric analysis

Plant development was assessed through biometric measurements, including tree height, canopy diameter, canopy projected area, canopy volume, and the height-to-canopy diameter ratio. Tree height was measured from the soil surface to the highest point of the canopy. Canopy length and width were measured in two perpendicular directions, and the average canopy diameter was calculated as:


$$D=\;\frac{L+W}{2}$$

Where *D* is the average canopy diameter, *L* is canopy length, and *W* is canopy width.

Canopy projected area was estimated assuming an elliptical canopy projection:


$$A=\;\pi \;x\;\frac{L}{2}\;x\;\frac{W}{2}$$

Where *A* is canopy area, *L* is canopy length, and *W* is canopy width

Canopy volume was estimated using an ellipsoidal approximation:


$$V=\;\frac{\pi }{6}\;x\;H\;x\;L\;x\;W$$

Where *V* is canopy volume and *H* is tree height.

The height-to-canopy diameter ratio was calculated to characterize canopy architecture:


$$H:D\;ratio=\;\frac{H}{D}$$

Linear measurements were expressed in meters, canopy area in m², and canopy volume in m³.

### 2.5 Fruit yield and juice quality analysis

Fruit yield was determined by harvesting and weighing all fruit from the ten central trees within each plot. Yield per tree was calculated and then converted to a hectare basis using the planting density of 717 trees ha^-1^. During harvest, a representative fruit subsample was collected from each plot for juice quality analysis. Fruit samples were processed at the Citrus Research and Education Center Pilot Plant in Lake Alfred, Florida.

Juice was extracted using a Pin-Point Extractor (Model 091B Series; JBT Corporation, Lakeland, FL, USA), and juice weight was recorded. Soluble solids concentration, expressed as °Brix, was determined based on Archimedes’ principle by comparing the weight of a plummet in water with its weight in orange juice. Titratable acidity (TA) was determined by titration with sodium hydroxide (NaOH) to an endpoint pH of 8.2, using phenolphthalein as an indicator. TA was calculated based on the volume and normality of NaOH consumed and expressed as a percentage of citric acid. The °Brix-to-acid ratio was calculated as the ratio of °Brix to TA.

### 2.6 Partial factor productivity

Partial factor productivity (PFP) was calculated to assess nutrient-use efficiency under the different fertilization strategies. PFP was determined for N, P expressed as P_2_O_5_, and K expressed as K_2_O by dividing fruit yield by the corresponding nutrient application rate:


$$PFP=\;\frac{Fruityield\;(kg\;ha^{-1})}{Nutrientapplied\;(kg\;ha^{-1})}$$

Accordingly, PFP-N, PFP-P_2_O_5_, and PFP-K_2_O were expressed as kilograms of fruit produced per kilogram of N, P_2_O_5_, or K_2_O applied, respectively $\left({\text{kg fruit kg}}^{-1}\text{ nutrient}\right)$. Nutrient application rates were calculated on a hectare basis for each fertilization treatment, as described in the experimental design section.

### 2.7 Regression-based nutrient association analysis

Multivariate Ridge regression was performed using data from the 2025 and 2026 growing seasons to assess the relative associations between leaf nutrient concentrations and yield and juice quality variables within each fertilization protocol. Response variables were analyzed separately and included fruit yield, juice content per box, titratable acidity, total soluble solids expressed as °Brix, and the °Brix-to-acid ratio, calculated as the ratio of °Brix to titratable acidity.

Leaf nutrient concentrations were used as predictor variables. Macronutrients, including N, P, K, Ca, Mg, and S, were modeled separately from micronutrients, including B, Zn, Mn, Fe, and Cu. Models were fitted independently for the Standard, CRF1, and CRF2 fertilization programs, enabling the relative contribution of each nutrient to be evaluated within each fertilization strategy. Each fertilization-specific model included 16 plot-year observations, corresponding to eight experimental plots evaluated in both the 2025 and 2026 growing seasons. Because observations from the two years originated from the same experimental plots, the dataset comprised eight experimental units repeatedly evaluated over time rather than 16 independent experimental replicates.

Prior to model fitting, data were summarized within each fertilization protocol by calculating the number of observations, mean, standard deviation, and range for each response variable. This preliminary inspection was conducted to confirm that yield and juice quality variables exhibited sufficient variation within each protocol. Ridge regression was selected because leaf nutrient concentrations are commonly intercorrelated, and including multiple nutrients simultaneously in conventional multiple linear regression can result in multicollinearity and unstable coefficient estimates.

Ridge regression is a regularized regression method that applies an L2 penalty to shrink coefficient estimates, thereby improving coefficient stability when predictors are correlated or nonorthogonal (Hoerl and Kennard, 1970). Before analysis, both predictor and response variables were standardized using z-score transformation. Consequently, the resulting coefficients represent standardized Ridge coefficients, allowing direct comparison of the direction and relative magnitude of nutrient associations within each fertilization protocol.

The regularization parameter α was selected automatically using RidgeCV. A total of 200 candidate α values were tested on a logarithmic scale from 10^–6^ to 10^1^. For each combination of fertilization protocol, nutrient group, and response variable, the optimal α value was selected and used to estimate standardized coefficients. Thus, separate Ridge regression models were fitted for macro- and micronutrients and for each response variable.

Standardized coefficients were organized into matrices and visualized as heatmaps. The color scale was centered at zero for each response variable and separately for macro- and micronutrient models. Positive coefficients indicate positive associations between a given leaf nutrient and the response variable, whereas negative coefficients indicate inverse associations, after accounting for the simultaneous contribution of the other nutrients included in the model. Color intensity reflects the relative magnitude of the standardized coefficient.

Because Ridge regression was used as an exploratory association approach and the number of experimental units within each fertilization program was limited, coefficient magnitude and direction were interpreted cautiously. The standardized coefficients were therefore considered experiment-specific indicators of relative nutrient-response associations rather than causal effects or broadly generalized predictive relationships.

### 2.8 Statistical analysis

Data were initially evaluated for normality and homoscedasticity. Subsequently, each response variable was analyzed by analysis of variance (ANOVA) using the F-test, with fertilization treatment included as the main fixed effect and block included as the blocking factor, consistent with the randomized complete block design. When significant treatment effects were identified, treatment means were separated using Tukey’s honest significant difference (HSD) test at a 5% significance level. Because the experiment was conducted over multiple years, statistical analyses were performed separately by year; therefore, year effects were not interpreted as treatment effects. Partial factor productivity indices for N, P_2_O_5_, and K_2_O were analyzed using the same ANOVA structure.

To formally evaluate temporal variation and the stability of fertilization program responses across years, an additional across-year analysis was performed. For soil properties, leaf nutrient concentrations, tree biometric variables, fruit yield, and juice-quality variables, linear mixed-effects models were fitted with fertilization program, year, and fertilization program × year as fixed effects. Block and experimental plot were included as random effects to account for the randomized block design and the repeated evaluation of the same experimental plots over time. The significance of fertilization program, year, and their interaction was evaluated using Wald tests. These analyses included the 2024, 2025, and 2026 production years when measurements were available.

Because PFP indices were available only for 2025 and 2026 and the corresponding mixed-effects models resulted in a singular random-effects covariance structure, temporal responses of PFP-N, PFP-P_2_O_5_, and PFP-K_2_O were evaluated using generalized estimating equations (GEE). Fertilization program, year, and fertilization program × year were included as fixed effects, experimental plot was specified as the grouping unit, and an exchangeable working correlation structure was used to account for repeated observations of the same plots between years. Wald tests were used to assess the significance of the main effects and their interaction. Year-specific Tukey comparisons were retained to facilitate interpretation of differences among fertilization programs within each year.

Principal component analysis (PCA) was conducted to examine multivariate relationships among fertilization treatments and measured soil, leaf, biometric, yield, juice quality, and nutrient-use efficiency variables. Prior to PCA, all variables were standardized to zero mean and unit variance. Hierarchical cluster analysis (HCA) was performed using Euclidean distance as the dissimilarity metric and single-linkage clustering as the agglomeration method. PCA and HCA were performed separately for the 2025 and 2026 growing seasons to avoid confounding seasonal variation with treatment effects. HCA was used to identify similarity patterns among fertilization treatments based on the integrated set of response variables.

All statistical analyses were performed using Python 3.9.7. Data handling, statistical analyses, regression modeling, and visualization were conducted using pandas, NumPy, SciPy, statsmodels, scikit-learn, matplotlib, and seaborn.

## 3 Results

### 3.1 Effect of treatments had limited effects on soil chemical properties and macronutrient availability

Overall, fertilization treatments had limited effects on soil macronutrient availability, and most differences were restricted to specific years and soil depths (supplemental materials 1 and 2). No consistent treatment effect was observed across the experimental period for soil macronutrients, except for a few isolated responses. In 2026, soil P at the 20–40 cm depth was higher under the Standard treatment than under CRF2, while CRF1 did not differ from either treatment. In the same year, soil K at the 0–20 cm depth was higher under CRF1 than under CRF2, with Standard showing intermediate values. Although ANOVA indicated a significant treatment effect for soil Ca at the 0–20 cm depth in 2024, no differences among treatments were detected by Tukey’s test. At the 20–40 cm depth in 2024, soil Ca was higher under Standard than under CRF1, whereas CRF2 did not differ from either treatment. In 2026, soil Ca was greater at the 0–20 cm depth under CRF1 than under both Standard and CRF2. A similar pattern was observed for Mg, with Standard showing greater Mg than CRF1 at the 0–20 cm depth in 2025, while CRF2 was intermediate. In 2026, Mg at the 0–20 cm depth was greater under CRF1 than under Standard and CRF2. Soil S differed only at the 0–40 cm depth in 2026, when CRF2 showed lower values than Standard and CRF1 (**Supplementary Material 1**).

Soil chemical properties were also only slightly affected by fertilization treatments. Significant differences were observed for pH, organic matter, and cation exchange capacity (CEC), but these responses were restricted to specific years and depths. Soil pH differed only at the 0–20 cm depth in 2026, when CRF1 showed higher pH than Standard, while CRF2 did not differ from either treatment. Organic matter differed at the 0–20 cm depth in 2025 and 2026. In 2025, CRF1 showed lower organic matter than Standard and CRF2, whereas in 2026, CRF1 showed higher organic matter than CRF2, with Standard showing intermediate values. CEC differed only at the 0–20 cm depth in 2026, when CRF1 showed higher values than Standard and CRF2 (**Supplementary Material 2**).

### 3.2 Variable effects of fertilizer treatments on leaf macro- and micronutrient concentrations

Fertilization effects were observed only in some evaluation years on leaf macronutrient concentrations, with significant differences detected mainly in 2024 and 2026, whereas no treatment effects were observed for any macronutrient in 2025 (**Figure 3**) . In 2024, leaf N concentration was highest in CRF1, followed by Standard and CRF2 (**Figure 3A**). However, no treatment effects in leaf N were observed in 2025 or 2026 (**Figures 3B, C**).

**Figure 3:**
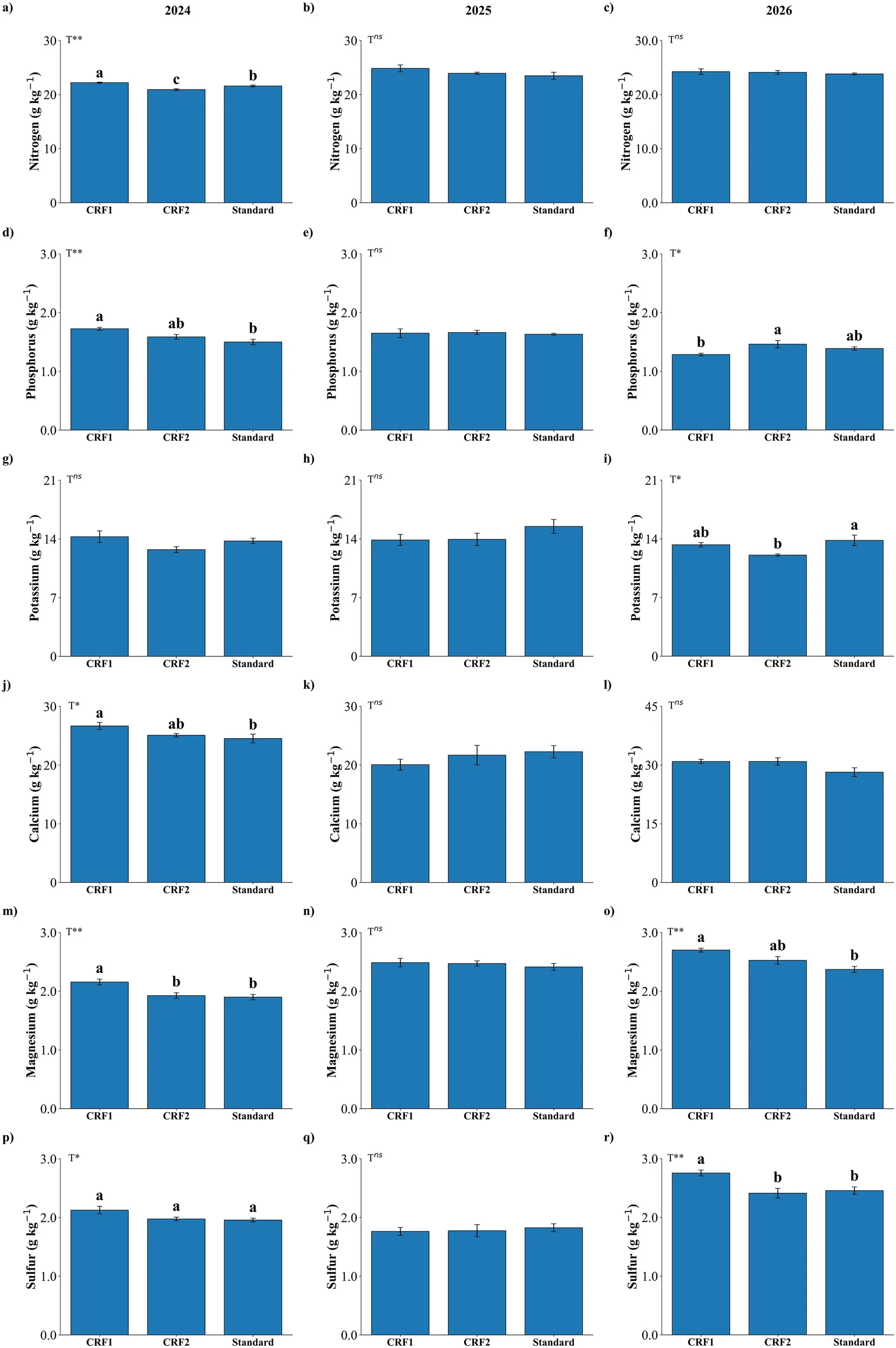
Leaf macronutrient concentrations of mature HLB-affected ‘Valencia’ sweet orange trees subjected to three fertilization programs (Standard, CRF1, and CRF2) during the 2024, 2025, and 2026 growing seasons. Panels show nitrogen (N; **A–C**), phosphorus (P; **D–F**), potassium (K; **G–I**), calcium (Ca; **J–L**), magnesium (Mg; **M–O**), and sulfur (S; **P–R**). Bars represent means ± standard error (n = 8). Within each year, different lowercase letters indicate significant differences among fertilization programs according to Tukey’s HSD test at *P* ≤ 0.05. Asterisks indicate a significant overall fertilization-program effect by ANOVA, whereas ns indicates no significant effect.

Leaf P concentration differed among treatments in 2024 (**Figure 3D**) and 2026 (**Figure 3E**). In 2024, CRF1 showed higher P concentration than Standard, whereas CRF2 did not differ from either treatment. In contrast, in 2026, CRF2 showed higher P concentration than CRF1, while Standard showed intermediate values and did not differ from the other treatments (**Figure 3F**). Leaf K concentration was affected only in 2026, when Standard showed higher values than CRF2, with CRF1 showing intermediate values (**Figure 3I**).

Leaf Ca concentration differed only in 2024, with CRF1 showing higher values than Standard, while CRF2 did not differ from either treatment (**Figure 3J**). Leaf Mg concentration was affected in both 2024 (**Figure 3M**) and 2026 (**Figure 3O**). In 2024, CRF1 showed higher Mg concentration than CRF2 and Standard. In 2026, CRF1 showed higher Mg concentration than Standard, whereas CRF2 showed intermediate values (**Figure 3O**). Leaf S concentration showed a significant treatment effect by ANOVA in 2024; however, no differences among treatments were detected by Tukey’s test (**Figure 3P**). In 2026, CRF1 resulted in higher leaf S concentration than both CRF2 and Standard (**Figure 3R**).

Leaf micronutrient concentrations were less responsive to fertilization treatments than macronutrients, with significant effects restricted to Mn, Fe, and Cu in specific evaluation years (**Figure 4**). No treatment effects were observed for leaf B or Zn concentrations in any year (**Figures 4A–F**). Leaf Mn concentration differed only in 2025, when CRF1 showed higher values than CRF2, whereas Standard did not differ from either treatment (**Figure 4H**). Leaf Fe concentration was affected in 2025 and 2026. In 2025, Standard resulted in higher Fe concentration than CRF1 and CRF2 (**Figure 4K**). In contrast, in 2026, CRF2 showed higher Fe concentration than Standard, while CRF1 showed intermediate values and did not differ from either treatment (**Figure 4L**). Leaf Cu concentration differed only in 2025, when Standard showed higher values than CRF2, whereas CRF1 did not differ from either treatment (**Figure 4N**).

**Figure 4:**
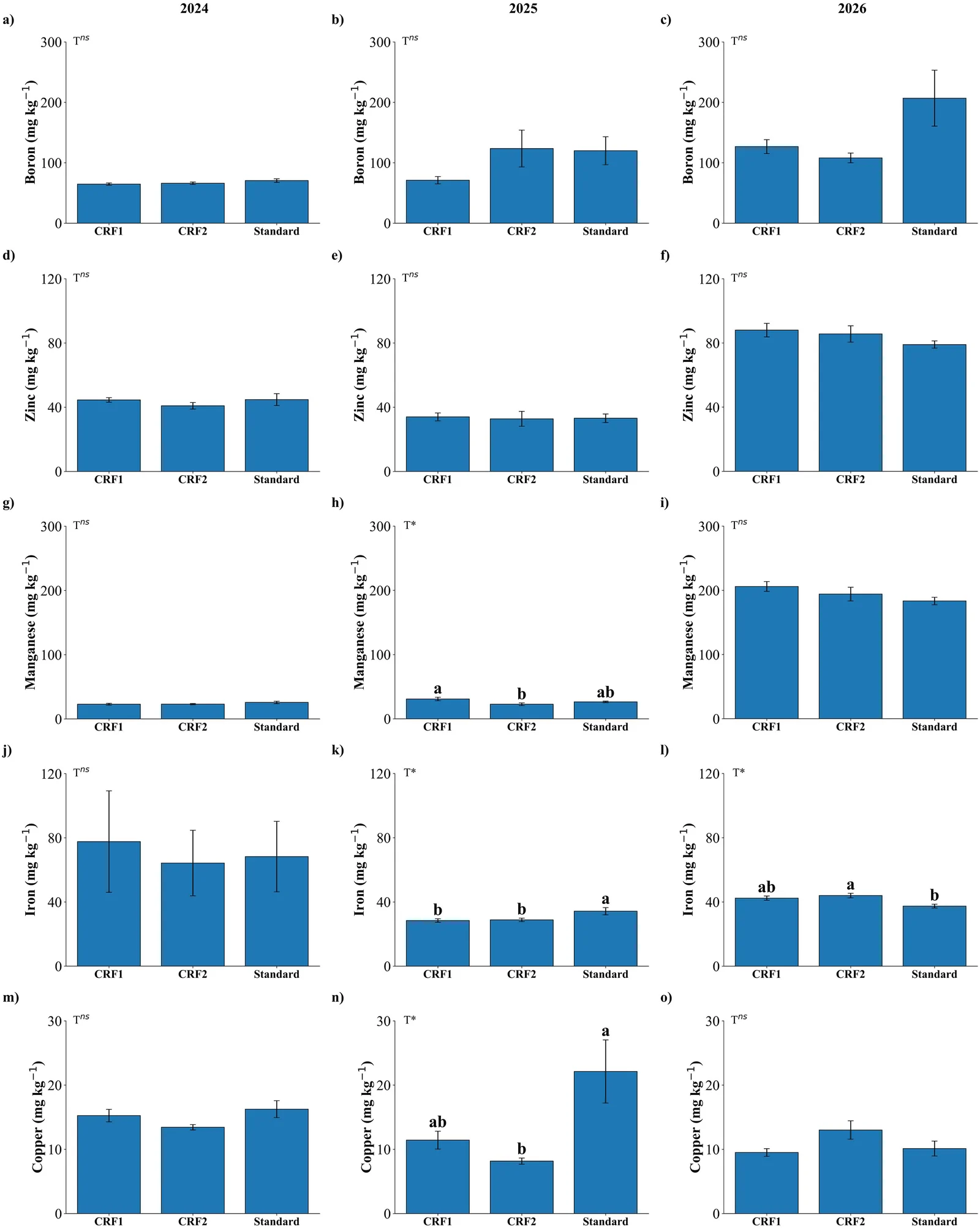
Leaf micronutrient concentrations of mature HLB-affected ‘Valencia’ sweet orange trees subjected to three fertilization programs (Standard, CRF1, and CRF2) during the 2024, 2025, and 2026 growing seasons. Panels show boron (B; **A–C**), zinc (Zn; **D–F**), manganese (Mn; **G–I**), iron (Fe; **J–L**), and copper (Cu; **M–O**). Bars represent means ± standard error (n = 8). Within each year, different lowercase letters indicate significant differences among fertilization programs according to Tukey’s HSD test at *P* ≤ 0.05. Asterisks indicate a significant overall fertilization-program effect by ANOVA, whereas ns indicates no significant effect.

### 3.3 Effects of fertilizer treatments are limited on canopy size, fruit yield, and juice quality but altered partial factor productivity

Biometric and canopy size variables were only slightly affected by fertilization treatments, with most variables showing no significant differences among treatments across the evaluation period (**Supplementary Material 3**). Tree height, canopy diameter, and canopy volume were not affected by fertilization treatments in any evaluation year, probably because these were bearing and mature trees.

Canopy area differed only in 2026, when Standard showed higher values than CRF2, whereas CRF1 did not differ from either treatment. Treatment effects on canopy size were observed only in 2024. The height-to-diameter ratio was higher under Standard than under CRF1 and CRF2, while the skewness index was higher under Standard than under CRF1, with CRF2 showing intermediate values. No treatment effects were observed for these canopy size variables in 2025 or 2026.

Fruit yield and juice quality variables showed limited responses to fertilization treatments, with no significant effects observed for most variables across the evaluation period (**Figure 5**). Fruit yield was not affected by fertilization treatments in 2024 (**Figure 5A**) or 2025 (**Figure 5B**). However, in 2026, CRF2 resulted in lower fruit yield than CRF1 and Standard, which did not differ from each other (**Figure 5C**). No significant treatment effects were observed for juice content per box (**Figures 4D–F**), titratable acidity (**Figures 5G-I**, soluble solids concentration expressed as °Brix (**Figures 5J-L**), or the °Brix-to-acid ratio in any evaluation year (**Figures 5M–O**).

**Figure 5:**
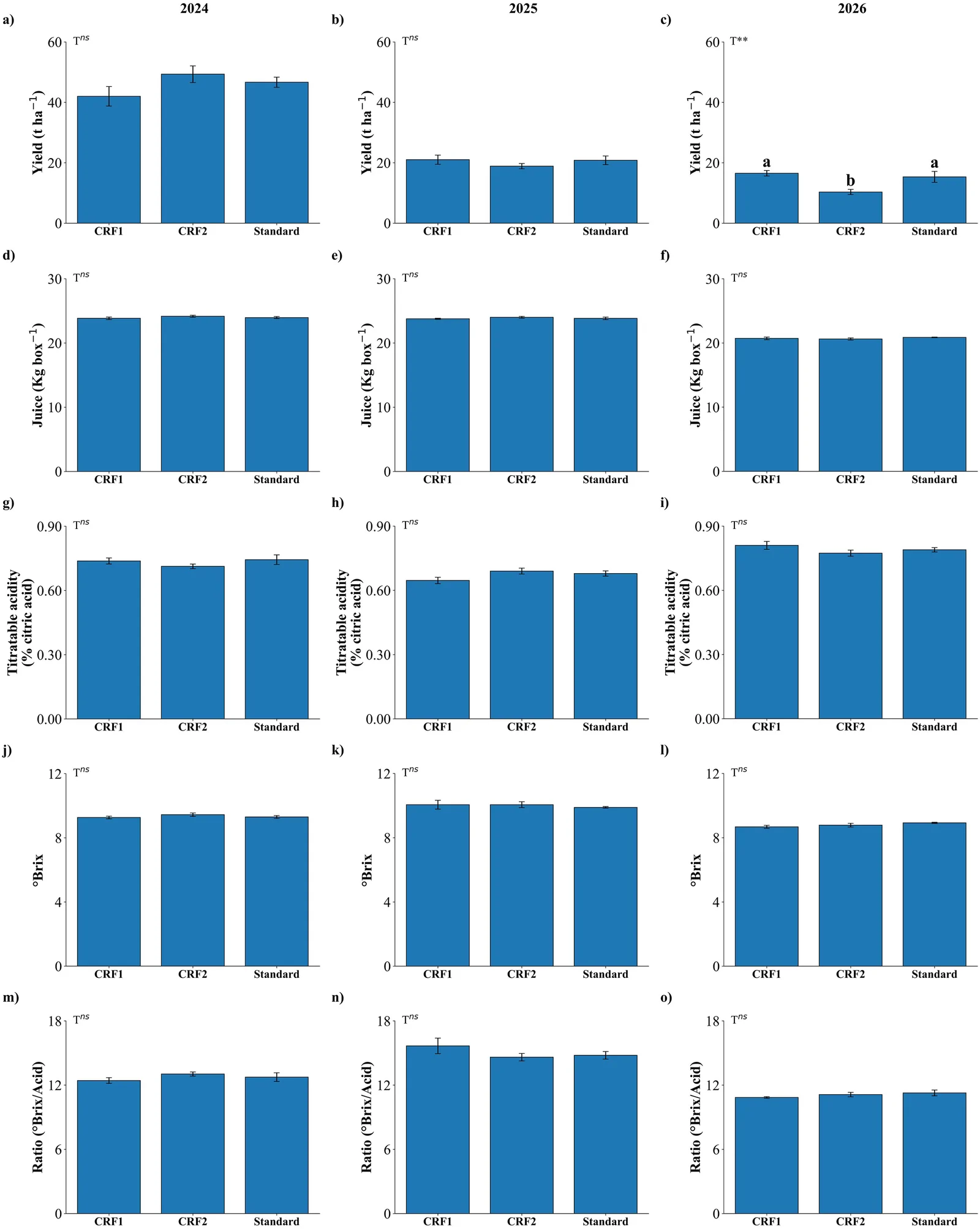
Fruit yield and juice-quality attributes of mature HLB-affected ‘Valencia’ sweet orange trees subjected to three fertilization programs (Standard, CRF1, and CRF2) during the 2024, 2025, and 2026 growing seasons. Panels show fruit yield **(A–C)**, juice content per box **(D–F)**, titratable acidity **(G–I)**, soluble solids concentration expressed as °Brix **(J–L)**, and °Brix-to-acid ratio **(M–O)**. Bars represent means ± standard error (n = 8). Within each year, different lowercase letters indicate significant differences among fertilization programs according to Tukey’s HSD test at *P* ≤ 0.05. Asterisks indicate a significant overall fertilization-program effect by ANOVA, whereas ns indicates no significant effect.

Partial factor productivity was affected by fertilization treatments, but the response varied according to the nutrient and evaluation year (**Figure 6**). For N, PFP was affected only in 2025, when Standard and CRF2 showed higher values than CRF1 (**Figure 6A**). In 2026, no significant differences among fertilization treatments were observed for PFP-N (**Figure 6B**).

**Figure 6:**
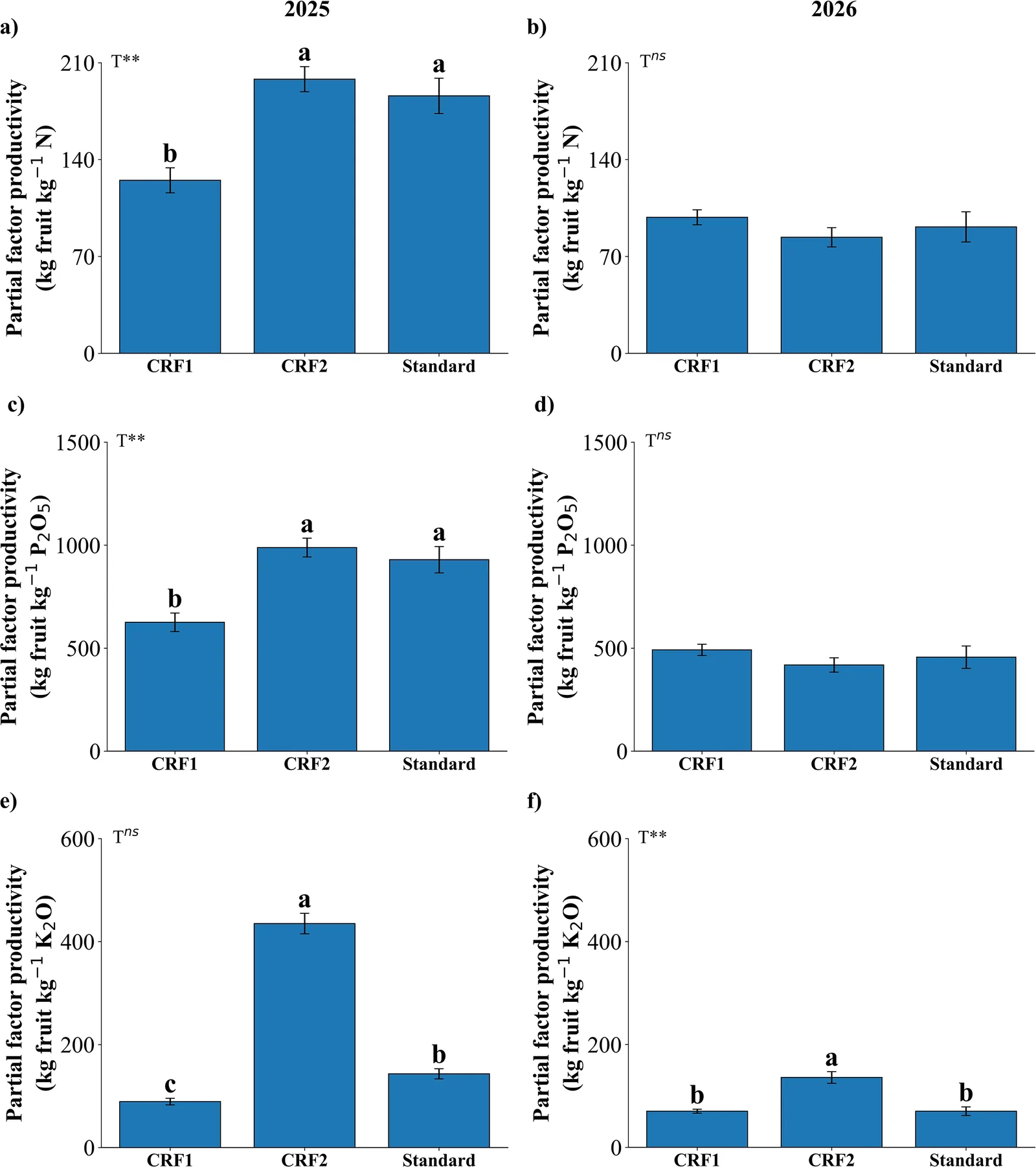
Partial factor productivity (PFP) of nitrogen, phosphorus, and potassium in mature HLB-affected ‘Valencia’ sweet orange trees subjected to three fertilization programs (Standard, CRF1, and CRF2) during the 2025 and 2026 growing seasons. Panels show PFP-N **(A, B)**, PFP-P_₂_O_₅_ **(C, D)**, and PFP-K₂O **(E, F)**, expressed as kilograms of fruit produced per kilogram of nutrient applied. Bars represent means ± standard error (n = 8). Within each year, different lowercase letters indicate significant differences among fertilization programs according to Tukey’s HSD test at *P* ≤ 0.05. Asterisks indicate a significant overall fertilization-program effect by ANOVA, whereas ns indicates no significant effect.

A similar pattern was observed for PFP-P_2_O_5_. In 2025, CRF1 showed lower PFP-P_2_O_5_ than Standard and CRF2, which did not differ from each other (**Figure 6C**). However, no treatment effect was observed for PFP-P_2_O_5_ in 2026 (**Figure 6D**).

In contrast, PFP-K_2_O differed among treatments in both evaluation years. In 2025, CRF2 showed the highest PFP-K_2_O, followed by Standard and then CRF1 (**Figure 6E**). In 2026, CRF2 also showed higher PFP-K_2_O than CRF1 and Standard, which did not differ from each other (**Figure 6F**).

### 3.4 Ridge regression reveals fertilization-specific associations between leaf nutrients and yield or juice quality

Ridge regression analysis revealed that the associations between leaf macronutrient concentrations and fruit yield or juice quality variables differed among fertilization programs (**Figure 7**). For fruit yield, the strongest positive associations were observed for leaf S under CRF2, leaf N under CRF2, and leaf P under CRF1 (**Figure 7A**). In contrast, leaf Ca under CRF2 showed the strongest negative association with yield, followed by negative associations of leaf Mg under CRF1 and leaf K under CRF2.

**Figure 7:**
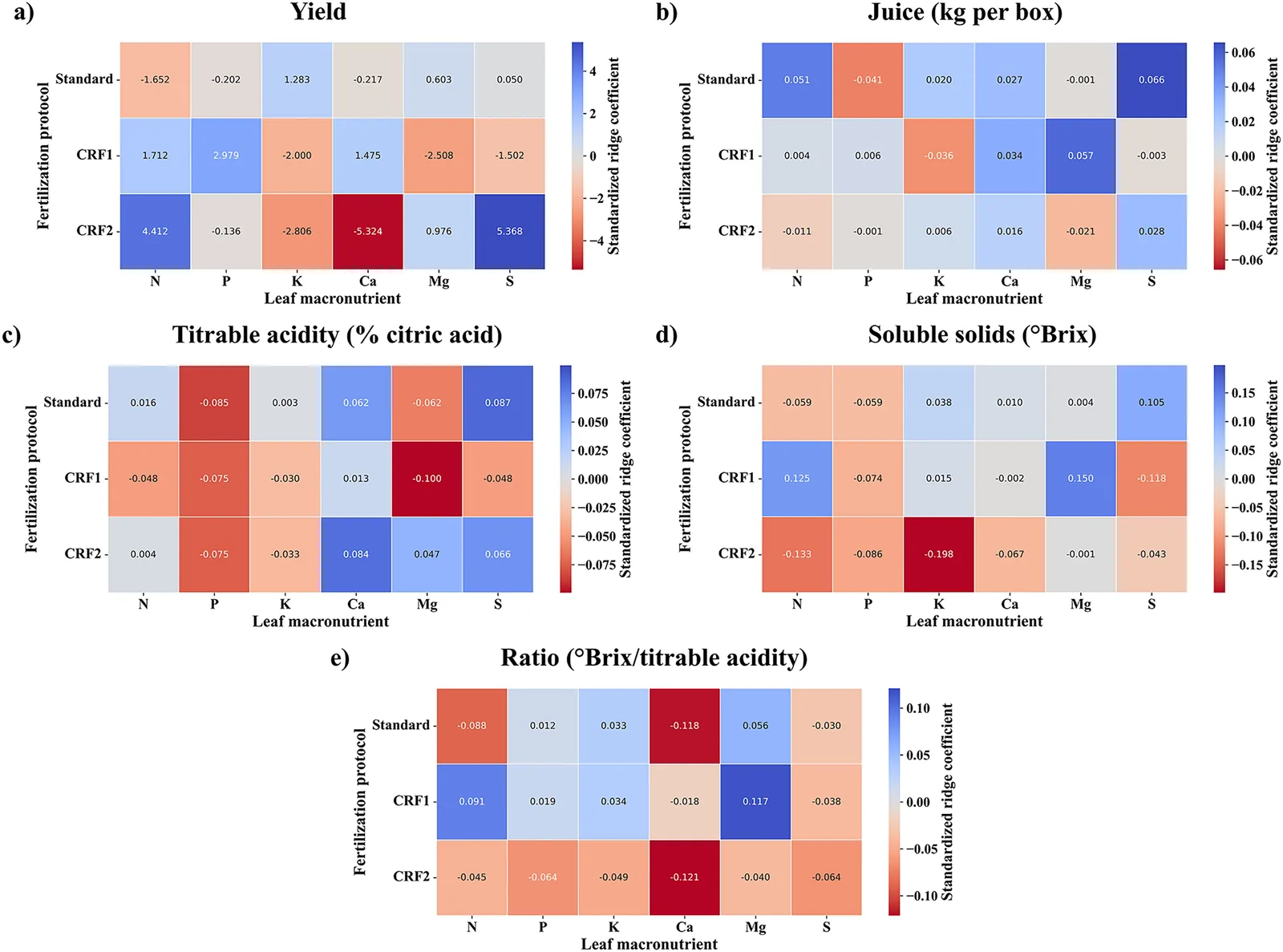
Standardized Ridge regression coefficients describing associations between leaf macronutrient concentrations and fruit yield or juice-quality variables in mature HLB-affected ‘Valencia’ sweet orange trees under the Standard, CRF1, and CRF2 fertilization programs. Panels represent fruit yield **(A)**, juice content per box **(B)**, titratable acidity **(C)**, soluble solids concentration expressed as °Brix **(D)**, and °Brix-to-acid ratio **(E)**. Predictor variables were leaf N, P, K, Ca, Mg, and S concentrations. Positive coefficients indicate positive associations and negative coefficients indicate inverse associations after accounting for the simultaneous contribution of the other macronutrients included in the model. Color intensity represents the magnitude of the standardized Ridge coefficient. Models were fitted separately for each fertilization program using observations from the 2025 and 2026 growing seasons.

Standardized Ridge coefficients for juice content per box were generally low compared with those observed for yield, indicating weaker macronutrient associations with this variable (**Figure 7B**). The highest positive coefficients were observed for leaf S under Standard and leaf Mg under CRF1, whereas negative associations were small and mainly observed for leaf P under Standard and leaf Mg under CRF2 (**Figure 7B**).

Titratable acidity was positively associated mainly with leaf S under Standard, leaf Ca under CRF2, and leaf Ca under CRF1 (**Figure 7C**). Conversely, leaf Mg under CRF1 and leaf P under Standard and CRF1 showed negative associations with titratable acidity. For soluble solids concentration, expressed as °Brix, positive associations were observed mainly for leaf Mg and N under CRF1 and leaf S under Standard, whereas leaf K under CRF2 showed the strongest negative association (**Figure 7D**).

Leaf Mg under CRF1 for the °Brix-to-acid ratio showed the strongest positive association, followed by leaf N under CRF1 and leaf Mg under Standard. Negative associations were mainly observed for leaf Ca under CRF2 and Standard, as well as for leaf N under Standard (**Figure 7E**). Overall, the Ridge regression results indicated that nutrient–response associations were nutrient program-specific, with no single macronutrient consistently associated with all fruit yield and juice quality variables across fertilization treatments.

Ridge regression analysis indicated that the associations between leaf micronutrient concentrations and fruit yield or juice quality also varied among fertilization programs (**Figure 8**). The strongest positive association for fruit yield was observed for leaf Zn under CRF2, followed by leaf Fe under CRF2 and Cu under CRF1. In contrast, leaf Mn under CRF2 showed the strongest negative association with yield, followed by negative associations of leaf B under CRF2 and leaf Mn under Standard (**Figure 8A**).

**Figure 8:**
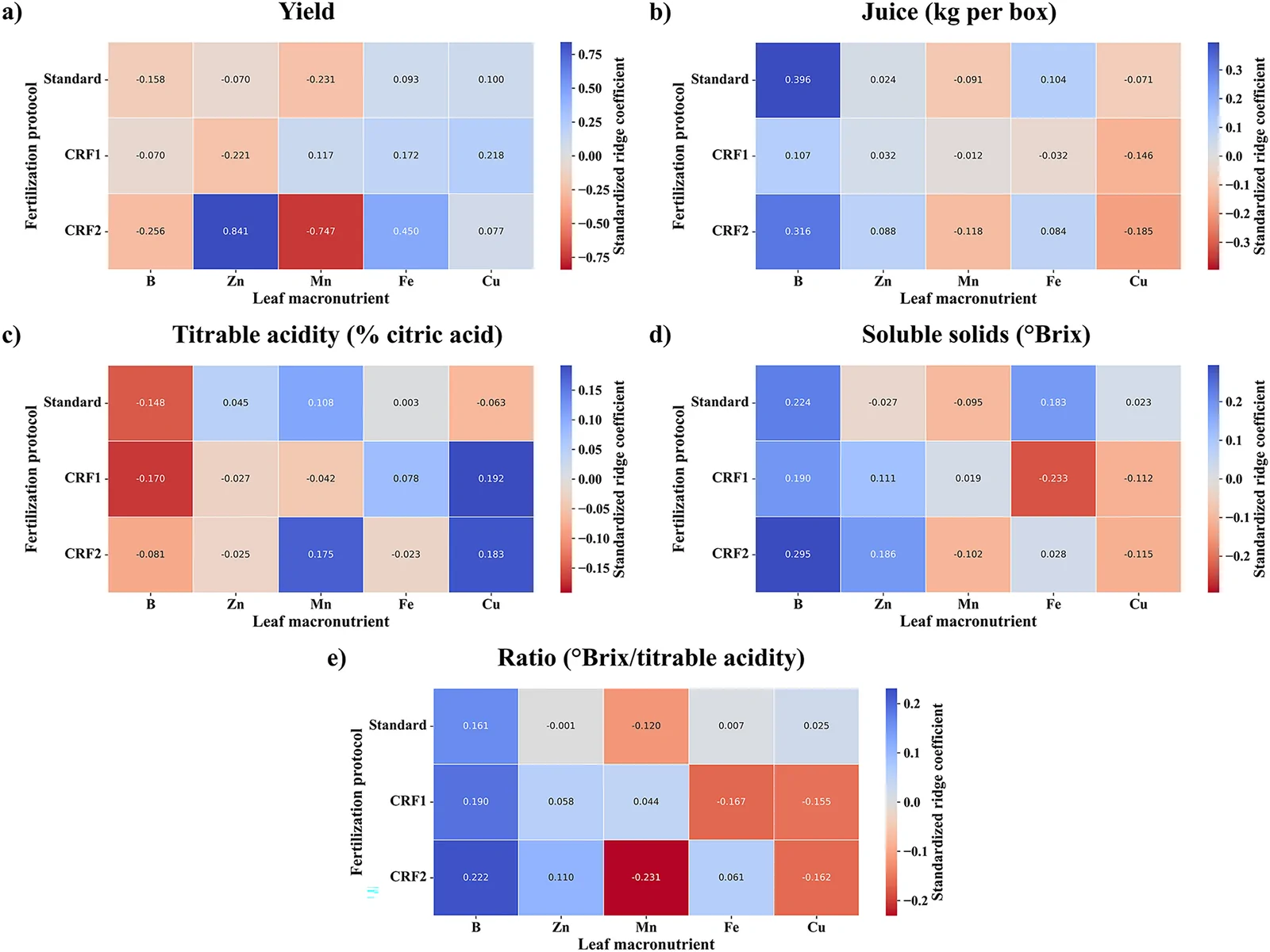
Standardized Ridge regression coefficients describing associations between leaf micronutrient concentrations and fruit yield or juice-quality variables in mature HLB-affected ‘Valencia’ sweet orange trees under the Standard, CRF1, and CRF2 fertilization programs. Panels represent fruit yield **(A)**, juice content per box **(B)**, titratable acidity **(C)**, soluble solids concentration expressed as °Brix **(D)**, and °Brix-to-acid ratio **(E)**. Predictor variables were leaf B, Zn, Mn, Fe, and Cu concentrations. Positive coefficients indicate positive associations and negative coefficients indicate inverse associations after accounting for the simultaneous contribution of the other micronutrients included in the model. Color intensity represents the magnitude of the standardized Ridge coefficient. Models were fitted separately for each fertilization program using observations from the 2025 and 2026 growing seasons.

Positive associations for juice content per box were mainly observed for leaf B under Standard and CRF2, whereas negative associations were generally weaker and were mainly related to leaf Cu under CRF2 and CRF1 and leaf Mn under CRF2 (**Figure 8B**). Titratable acidity showed positive associations mainly with leaf Cu under CRF1 and CRF2 and leaf Mn under CRF2, while leaf B showed negative associations across all fertilization programs (**Figure 8C**).

For soluble solids concentration, expressed as °Brix, leaf B showed positive associations across all fertilization programs, particularly under CRF2 and Standard. Leaf Zn also showed a positive association under CRF2. In contrast, leaf Fe under CRF1 and leaf Cu under CRF1 and CRF2 showed negative associations with °Brix (**Figure 8D**). For the °Brix-to-acid ratio, leaf B showed positive associations across all fertilization programs, especially under CRF2, whereas leaf Mn under CRF2 and leaf Cu under CRF1 and CRF2 showed negative associations (**Figure 8E**).

Overall, these results indicate that the associations between leaf micronutrients and fruit yield or juice quality were fertilization-protocol specific. As for macronutrients, these Ridge coefficients should be interpreted as exploratory associations rather than direct causal effects of individual micronutrients.

### 3.5 Temporal effects and fertilization program × year interactions

The analysis across years revealed that temporal variation contributed substantially to the responses of several soil, plant, and productivity variables (**Supplementary Table S4**). Significant year effects were detected for soil pH, CEC, K, Mg, and S at 0–20 cm and for soil pH, P, K, Ca, Mg, and S at 20–40 cm. Significant fertilization program × year interactions occurred for soil pH, organic matter, CEC, Ca, and Mg at 0–20 cm, whereas at 20–40 cm the interaction was significant only for soil P. These results indicate that the effects of the fertilization programs on selected soil properties were not constant throughout the experimental period.

Leaf nutrient concentrations were also strongly influenced by year. Significant year effects were observed for N, P, K, Ca, Mg, and S, as well as for B, Zn, Mn, and Cu, whereas Fe remained unaffected by year. Significant fertilization program × year interactions were detected for leaf P, Ca, S, B, and Cu, demonstrating that the magnitude of the nutritional response to the fertilization programs differed among production years for these nutrients. In contrast, most tree biometric variables showed no significant interaction between fertilization program and year, although canopy diameter and canopy area varied significantly among years and the skewness index showed both a fertilization program and a year effect.

Fruit yield was significantly affected by fertilization program (*P* = 0.014), year (*P* < 0.001), and the fertilization program × year interaction (*P* = 0.004; **Supplementary Table S4**; **Figure 9A**). Despite the strong decline in yield over the experimental period, no significant differences among fertilization programs were observed in either 2024 or 2025. In 2026, however, CRF2 resulted in significantly lower fruit yield than Standard and CRF1, whereas Standard and CRF1 did not differ from each other (**Figure 9A**). Thus, the significant fertilization program × year interaction reflects a change in the relative performance of the fertilization programs over time, with the lower yield under CRF2 becoming evident only in the final production year. Among juice-quality variables, year significantly affected juice content, titratable acidity, soluble solids, and the °Brix-to-acid ratio; however, a significant fertilization program × year interaction was observed only for titratable acidity (*P* = 0.028).

**Figure 9:**
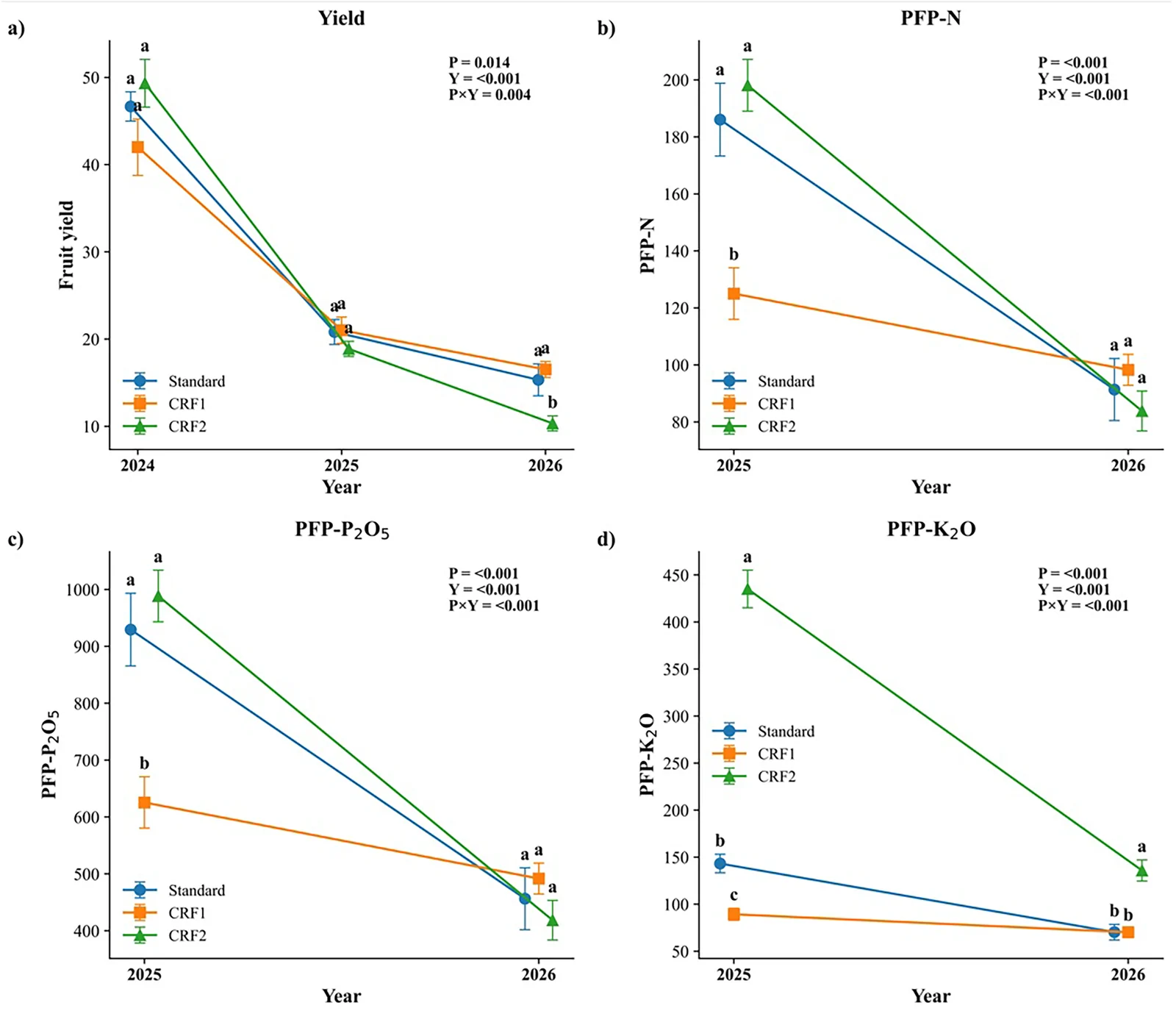
Temporal responses of fruit yield and nutrient partial factor productivity in mature HLB-affected ‘Valencia’ sweet orange trees subjected to the Standard, CRF1, and CRF2 fertilization programs. Panels show fruit yield **(A)**, PFP-N **(B)**, PFP-P_₂_O_₅_ **(C)**, and PFP-K_₂_O **(D)**. Fruit yield was evaluated from 2024 to 2026, whereas PFP indices were evaluated in 2025 and 2026. Symbols represent treatment means ± standard error (n = 8). Different lowercase letters within each year indicate significant differences among fertilization programs according to Tukey’s HSD test at *P* ≤ 0.05. *P*-values for fertilization program, year, and fertilization program × year interaction are shown in each panel. Temporal effects for fruit yield were evaluated using a linear mixed-effects model, whereas PFP indices were analyzed using generalized estimating equations to account for repeated observations over time.

Partial factor productivity showed particularly strong temporal dependence on fertilization program, with significant effects of fertilization program, year, and their interaction for PFP-N, PFP-P_2_O_5_, and PFP-K_2_O (all *P* < 0.001; **Supplementary Table S4**; **Figures 9B–D**). In 2025, PFP-N was significantly higher under Standard and CRF2 than under CRF1, whereas no significant differences among fertilization programs were observed in 2026 (**Figure 9B**). The same pattern was observed for PFP-P_2_O_5_, for which Standard and CRF2 showed higher values than CRF1 in 2025, while the three fertilization programs did not differ significantly in 2026 (**Figure 9C**). In contrast, PFP-K_2_O showed a more persistent response to fertilization program. In 2025, CRF2 had the highest PFP-K_2_O, followed by Standard and CRF1, whereas in 2026 CRF2 remained significantly higher than both Standard and CRF1, which did not differ from each other (**Figure 9D**). Therefore, the significant interactions for the three PFP indices indicate that the magnitude and, for PFP-N and PFP-P_2_O_5_, the statistical differentiation among fertilization programs changed between production years.

### 3.6 Hierarchical clustering revealed distinct fertilization response profiles, with strong separation based on yield and partial factor productivity

Hierarchical clustering analysis showed that fertilization programs generated distinct integrated response profiles based on leaf nutrient concentrations, fruit yield, juice quality, and partial factor productivity variables (**Figure 10**). In In both years, 2025 (**Figure 10A**) and 2026 (**Figure 10B**), CRF1 formed an isolated cluster, whereas CRF2 and Standard were grouped together, indicating greater similarity between these two fertilization programs based on the integrated response of leaf nutrient concentrations, fruit yield, juice quality, and partial factor productivity variables.

**Figure 10:**
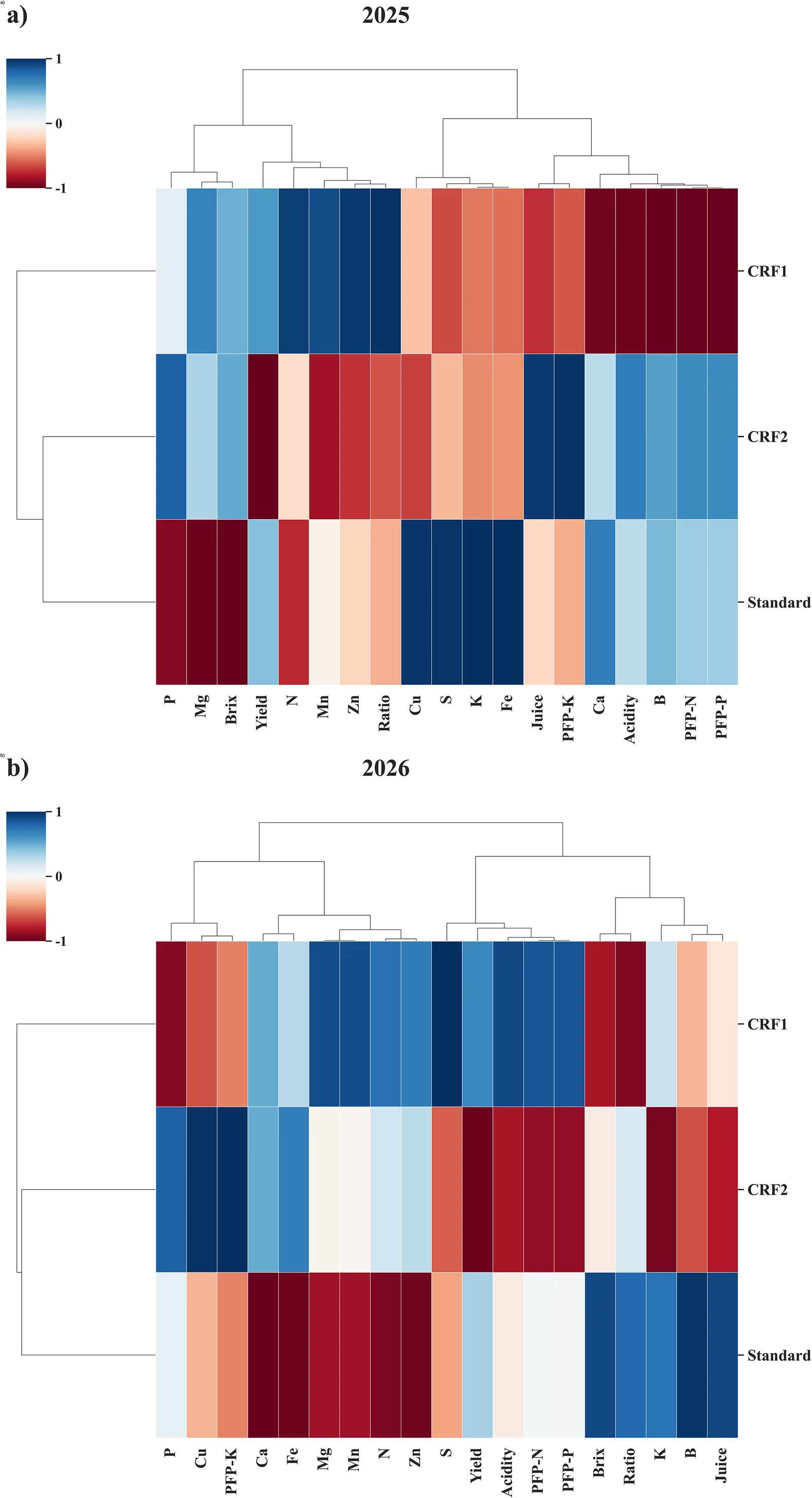
Hierarchical cluster analysis (HCA) of integrated nutritional, productive, juice-quality, and nutrient-productivity responses of mature HLB-affected ‘Valencia’ sweet orange trees subjected to the Standard, CRF1, and CRF2 fertilization programs in 2025 **(A)** and 2026 **(B)**. Variables included leaf macro- and micronutrient concentrations, fruit yield, juice-quality attributes, and partial factor productivity indices. Data were standardized using z-scores before analysis. Hierarchical clustering was performed using Euclidean distance and single-linkage clustering. The color scale represents standardized values, with positive and negative values indicating observations above and below the overall variable mean, respectively. Dendrograms illustrate similarities among fertilization programs and response variables.

In 2025, CRF1 was characterized by lower standardized values for PFP-N, PFP-P_2_O_5_, and PFP-K_2_O, but higher values for fruit yield (**Figure 10A**). In contrast, CRF2 showed high standardized values for PFP-K_2_O and juice production, as well as positive values for PFP-N and PFP-P_2_O_5_, but lower values for yield. The Standard protocol showed positive standardized values for PFP-N, PFP-P_2_O_5_, and yield, but lower values for PFP-K_2_O. In the variable clustering, fruit yield was grouped with leaf N, Mn, Zn, and the °Brix-to-acid ratio, whereas PFP-N and PFP-P_2_O_5_ clustered together. PFP-K_2_O was grouped with juice production, suggesting a close multivariate association between K_2_O productivity and juice yield-related performance in 2025.

In 2026, the multivariate pattern changed. CRF1 showed positive standardized values for PFP-N, PFP-P_2_O_5_, and yield, but lower values for PFP-K_2_O. CRF2 showed the opposite pattern, with lower standardized values for PFP-N, PFP-P_2_O_5_, and yield, but higher values for PFP-K_2_O (**Figure 10B**). The Standard protocol was characterized by higher standardized values for juice production and °Brix, together with slightly positive values for yield, but lower values for PFP-K_2_O. In 2026, yield clustered with acidity, PFP-N, and PFP-P_2_O_5_, suggesting that fruit production was more closely associated with N and P_2_O_5_ productivity than with K_2_O productivity in that evaluation year.

Overall, the HCA indicated that treatment separation was strongly influenced by yield and PFP variables. Although several individual variables showed limited responses in the univariate analyses, the integrated multivariate approach revealed clear differences among fertilization programs, particularly in the balance between fruit yield and nutrient productivity.

### 3.7 Multivariate analysis on the role of nutrient productivity and leaf nutrient status in treatment discrimination

Principal component analysis (PCA) was performed to integrate leaf nutrient concentrations, fruit yield, juice quality, and partial factor productivity variables in 2025 (**Figures 11A, C**) and 2026 (**Figures 10B, D**). In 2025, the first two principal components explained 40.2% of the total variation, with Dim1 and Dim2 accounting for 23.7% and 16.5%, respectively (**Figures 11A, C**). The variables with the highest contribution to the PCA structure were mainly associated with partial factor productivity, especially PFP-N and PFP-P_2_O_5_ (**Figure 11A**). In the biplot, the fertilization treatments showed partial overlap, indicating that their overall multivariate responses were not completely separated in 2025 (**Figure 10C**). However, CRF1 tended to be distributed toward the positive side of Dim1, in the direction of leaf N, °Brix, Mg, and the °Brix-to-acid ratio, whereas CRF2 and Standard showed greater dispersion toward variables associated mainly with PFP.

**Figure 11:**
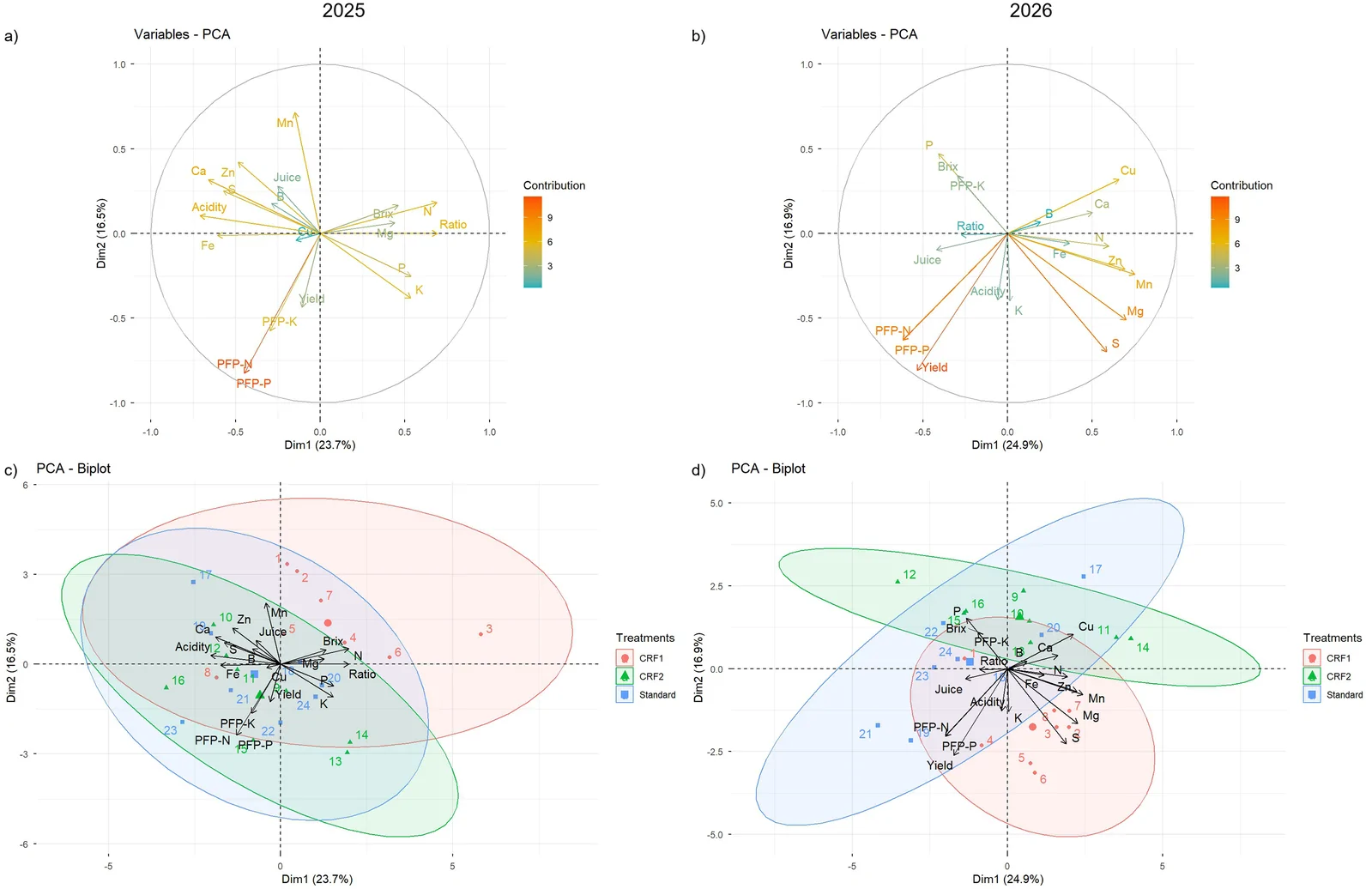
Principal component analysis (PCA) integrating leaf nutrient concentrations, fruit yield, juice-quality attributes, and partial factor productivity indices of mature HLB-affected ‘Valencia’ sweet orange trees under the Standard, CRF1, and CRF2 fertilization programs. Variable contribution plots are shown for 2025 **(A)** and 2026 **(B)**, and PCA biplots showing the distribution of experimental observations according to fertilization program are shown for 2025 **(C)** and 2026 **(D)**. In 2025, Dim1 and Dim2 explained 23.7% and 16.5% of the total variance, respectively, whereas in 2026 they explained 24.9% and 16.9%, respectively. Arrows indicate the direction and magnitude of variable loadings, and ellipses represent the distribution of observations associated with each fertilization program. All variables were standardized to zero mean and unit variance before PCA.

In 2026, the first two principal components explained 41.8% of the total variation, with Dim1 and Dim2 accounting for 24.9% and 16.9%, respectively (**Figures 10B, D**). The PCA contribution plot indicated that PFP-N, PFP-P_2_O_5_ and yield were among the variables contributing most to treatment discrimination (**Figure 11B**). In the biplot, treatment distribution was more visually differentiated than in 2025, although some overlap remained among the confidence ellipses (**Figure 10D**). CRF1 was mainly distributed toward the lower-right region of the biplot, in the direction of leaf Mg, S, Mn, Zn, and Fe. CRF2 showed greater dispersion toward the upper region of Dim2, in direction of Cu, P, °Brix and PFP-K, whereas Standard was more broadly distributed along the negative side of Dim1 and was associated with variables such as juice production, PFP-N, PFP-P_2_O_5_, and yield.

## 4 Discussion

### 4.1 Fertilizer programs produced limited changes in soil fertility but altered leaf nutritional status

The relatively small changes in soil chemical properties (**Supplementary Material 2**) and macronutrient availability (**Supplementary Material 1**) suggest that the fertilization programs did not consistently modify bulk soil fertility over the experimental period. This limited soil response is consistent with the characteristics of the experimental site, which contained approximately 945 g kg^-1^ sand and had low cation exchange capacity. Under these conditions, nutrients released into the soil solution may be rapidly displaced through the soil profile by rainfall and irrigation, retained only weaky by the soil exchange complex, or taken up by roots before substantial accumulation can be detected in bulk-soil samples. These processes are particularly relevante in Florida citrus soils, where high sand content, low nutrient-retention capacity, and recurrent rainfall and irrigation favor rapid nutrient movement through the root zone (Mohkam-Singh and Nunes, 2025). Moreover, under HLB endemic conditions, root decline and impaired nutrient acquisition may further weaken the association between soil nutrient availability and plant nutritional status (Atta et al., 2020a, 2023).

The release dynamics of the fertilizers may also help explain the apparent discrepancy between soil and leaf responses. Controlled-release fertilizers are designed to provide nutrients gradually over time rather than generating large and short-lived increases in soil nutrient concentrations, thereby improving the synchronization between nutrient supply and plant demand and potentially reducing nutrient losses (Govil et al., 2024; Jiang et al., 2024). Consequently, nutrients released from CRF granules may remain available for root uptake over an extended period without necessarily producing sustained increases in extractable nutrient concentrations in bulk soil. Recent studies have similarly shown that controlled-release fertilizers can prolong nutrient availability and improve nutrient uptake by regulating the temporal pattern of nutrient release (Ren et al., 2025; Xiao et al., 2026). This interpretation is especially relevant for CRF1, which increased leaf Mg and S concentrations in specific years despite only limited and transient effects on soil Mg and S. Thus, nutrient availability under CRF1 may have been more closely synchronized with root uptake and tree demand than reflected by nutrient accumulation in the sampled bulk soil.

Similar discrepancies between soil nutrient availability and plant nutritional responses have been reported in HLB-affected citrus. Atta et al. (2023) demonstrated that nutrient-management strategies altered leaf nutritional status and tree performance in mature HLB-affected ‘Valencia’ sweet orange, highlighting that plant response cannot be inferred solely from soil nutrient concentrations. Likewise (Hallman et al., 2023), reported that fertilizer application strategy influenced micronutrient uptake by severely HLB-affected citrus trees, whereas relationships between soil nutrient concentrations and plant uptake were not always consistent. More recently (Hallman et al., 2024), showed that increased soil organic matter enhanced N, P, and K uptake and modified citrus root-system development, further demonstrating that nutrient acquisition depends not only on the quantity of nutrients measured in soil but also on soil properties and root functionality.

Sampling scale and timing may have further contributed to the observed differences between soil and leaf responses. Soil analyses provide a snapshot of extractable nutrient availability at a specific sampling time and across the sampled soil volume, whereas nutrient availability in the rhizosphere can vary dynamically with fertilizer release, rainfall, irrigation, root activity, and plant demand. Therefore, transient changes in nutrient availability near fertilizer granules or active roots may not have been fully captured by the annual soil sampling conducted at the 0–20 and 20–40 cm depths. In contrast, leaf nutrient concentrations integrate nutrient acquisition, transport, and accumulation over a longer period and may therefore reveal nutritional responses that are not apparent from a single soil sampling event. This temporal component is particularly relevant for citrus, as recent field evidence has demonstrated pronounced seasonal variation in leaf nutrient concentrations in mature HLB-affected ‘Valencia’ sweet orange trees (Fajardo et al., 2025). In addition, comparisons among soil and plant-based nutritional diagnostic approaches in HLB-affected citrus have highlighted that different analytical compartments provide complementary information on tree nutrient status (Fox et al., 2025).

These mechanisms are particularly important under HLB conditions because the disease substantially affects fine-root development and nutrient acquisition (Atta et al., 2020a; Hallman et al., 2022). Consequently, treatment differences in leaf nutrient concentrations may reflect not only the amount of extractable nutrients present in bulk soil but also the timing and localization of nutrient release relative to active roots and the capacity of the root system to acquire those nutrients. Taken together, these findings indicate that limited changes in bulk-soil nutrient concentrations do not necessarily imply limited nutrient availability to the tree. Rather, the greater foliar response observed under CRF1 likely reflects the combined effects of fertilizer composition, nutrient-release dynamics, soil characteristics, and root-mediated nutrient acquisition. These results reinforce the importance of integrating soil and plant tissue analyses when evaluating fertilization strategies for mature HLB-affected citrus.

### 4.2 Fertilizer programs differed in the balance between fruit yield and K_2_O productivity

Fruit yield was relatively stable across fertilization programs during the first two evaluation years (**Figure 5**). CRF1 maintained yields comparable to those obtained under the conventional Standard program, whereas CRF2 did not sustain the same level of productive performance in the final evaluation year. However, the comparable yield performance of CRF1 should not be interpreted as evidence of overall superiority, because this program also supplied greater nutrient inputs and consequently showed lower PFP for some nutrients. Thus, the agronomic performance of CRF1 should be interpreted in the context of both sustained fruit production and nutrient input requirements. The divergence between CRF1 and CRF2 may reflect differences in total nutrient input, fertilizer composition, and nutrient-release dynamics. In HLB-affected citrus trees, root decline and reduced nutrient uptake capacity can decrease the predictability of yield responses to fertilization, even when fertilizer programs are designed to enhance nutrient availability. Therefore, tree performance under HLB depends on nutrient supply and on the ability of compromised root systems to acquire, translocate, and use those nutrients. This is particularly relevant because nutrient supply can influence root growth and lifespan in HLB-affected citrus trees (Atta et al., 2020a).

The fruit yield reduction observed under CRF2 in 2026 is particularly noteworthy because this treatment also exhibited lower leaf K concentrations than the Standard treatment in the same year (**Figure 4I**). Potassium is a key nutrient in citrus production, contributing to carbohydrate transport, fruit growth, yield formation, fruit size, and multiple fruit quality attributes (Wu et al., 2021b). Accordingly, adequate K management is essential for optimizing citrus productivity and fruit quality, especially because fertilization responses depend on nutrient balance, tree demand, and environmental conditions (Alva et al., 2006). In addition, citrus yield and nutrient-use efficiency are shaped by interactions between N and K fertilization and water-management strategy, indicating that nutrient-input efficiency does not necessarily increase proportionally with fertilizer rate (Quaggio et al., 2019). Together, these results suggest that the lower yield under CRF2 may have been associated with an inadequate nutritional balance, particularly in relation to K_2_O supply. Differences in nutrient release dynamics and synchronization between nutrient availability and tree demand may also have contributed to this response; however, these mechanisms were not directly evaluated in the present study and should therefore be considered hypotheses requiring further investigation.

Juice quality attributes were not significantly affected by fertilization program, including juice content, titratable acidity, °Brix, and the °Brix-to-acid ratio (**Figure 6**). This response suggests that the contrasting fertilizer programs had a greater influence on fruit yield than on internal fruit quality. However, citrus fruit-quality responses to nutrient management can vary substantially according to nutrient supply and production conditions. For example, Luo et al. (2025) reported that a 50% reduction in P fertilizer input in mature pomelo trees es maintained fruit yield while increasing lycopene and vitamin C concentrations, indicating that fruit quality may respond positively to optimized nutrient inputs rather than to greater fertilizer supply. Similar variability have been observed in other citrus fertilization studies, where changes in nutrient management affected yield or nutrient-use efficiency more consistently than fruit quality traits, depending on nutrient rate, water supply, and production system (Quaggio et al., 2019; Esteves et al., 2021; Basar et al., 2025). Therefore, under the conditions of the present study, differences among the Standard, CRF1, and CRF2 programs were expressed primarily through yield and nutrient productivity rather than through juice technological attributes.

Partial factor productivity provided a complementary perspective on the agronomic performance of each fertilization program. In 2025, PFP-N and PFP-P_2_O_5_ were greater under the Standard and CRF2 programs than under CRF1 (**Figure 8**). However, because fruit yield did not differ among treatments in that year, the lower PFP-N and PFP-P_2_O_5_ values under CRF1 were likely attributable to its greater nutrient input rather than to reduced tree productivity. Therefore, although CRF1 produced less fruit per unit of N or P_2_O_5_ applied in 2025, this lower apparent nutrient productivity did not indicate a yield penalty. This result highlights the importance of interpreting nutrient-use efficiency indices, including PFP, in conjunction with yield, fertilizer input, and management context, because these indices integrate both crop productivity and nutrient application rate while representing only one component of overall system performance (Fixen et al., 2015).

The most pronounced difference among fertilization programs was observed for PFP-K_2_O (**Figure 6**). CRF2 exhibited the highest PFP-K_2_O in both 2025 and 2026, indicating greater fruit production per unit of K_2_O applied. However, because PFP is mathematically dependent on both fruit yield and nutrient application rate, higher PFP-K_2_O under CRF2 should not be interpreted as direct evidence of superior nutrient-use efficiency or agronomic performance. In particular, the loser K_2_O input under CRF2 contributed to its higher PFP-K_2_O values. This distinction is especially important in 2026, when CRF2 exhibited the higher PFP-K_2_O despite producing lower fruit yield than Standard and CRF1. Thus, the higher PFP-K_2_O under CRF2 primarily reflects greater apparent productivity per unit of K_2_O rather than unequivocally greater K-use efficiency.

Collectively, these results demonstrate a clear contrast between the two CRF programs. CRF1 maintained yields comparable to those obtained under the Standard program and generated a distinct foliar nutritional profile, although its higher nutrient input reduced PFP in some cases. Conversely, CRF2 improved apparent K_2_O productivity, but this apparent advantage was accompanied by reduced yield in 2026. Therefore, PFP should not be interpreted as a stand-alone indicator when comparing fertilization programs. In mature HLB-affected citrus trees, the most effective fertilization strategy is not necessarily the one that maximizes PFP, but rather the one that sustains yield while promoting balanced leaf nutrition and efficient nutrient use.

The across-year analysis confirmed that fruit yield was strongly influenced by temporal variation and that the relative performance of the fertilization programs was not stable across the experimental period. The significant fertilization program × year interaction indicates that treatment differences depended on production year rather than representing a consistent response across seasons. Although the three fertilization programs produced comparable yields in 2024 and 2025, CRF2 showed a significant yield reduction relative to Standard and CRF1 in 2026. This temporal divergence reinforces that fertilizer-program performance in mature HLB-affected citrus should be evaluated across multiple production cycles rather than inferred from a single season. The overall decline in yield across years was also significant and likely reflects the combined influence of tree condition and environmental stresses during the study, including Hurricane Milton in October 2024 and chilling/freezing conditions during the 2024–2025 and 2025–2026 winters. Therefore, the year effect should be interpreted as temporal variation in the production environment rather than as an experimental treatment, whereas the significant fertilization program × year interaction demonstrates that the ability of the fertilization programs to sustain productivity differed across years.

### 4.3 CRF1 produced a distinct nutritional -productive profile compared with standard and CRF2

Hierarchical clustering indicated that CRF1 exhibited a distinct integrated nutritional and productive profile relative to the Standard and CRF2 treatments (**Figure 9**). This separation suggests that the two controlled-release fertilizer programs were not agronomically equivalent, despite both being CRF-based strategies. The distinct clustering of CRF1 likely reflects its broader nutrient composition and release dynamics, which resulted in a different combination of leaf nutrient concentrations, fruit yield, juice quality attributes, and partial factor productivity. This pattern supports the interpretation that citrus performance is influenced by nutrient balance and multi-nutrient interactions rather than by isolated nutrient concentrations, and fertilizer effects should be assessed through integrated nutritional profiles rather than single-nutrient responses alone, because multi-nutrient associations may identify productivity constraints in citrus more effectively than individual nutrient evaluations (Krug et al., 2023).

The PCA results further indicated that treatment differences were strongly associated with nutrient productivity indices and fruit yield. This finding is important because CRF1 and CRF2 differed not only in controlled-release fertilizer strategy but also in nutrient balance and total nutrient input. Thus, the multivariate separation among treatments likely reflects the combined influence of fertilizer composition, release dynamics, and nutrient rate on the relationship between leaf nutritional status and fruit production. Within this context, the higher PFP-K_2_O observed under CRF2 should be interpreted as an efficiency-related response rather than as direct evidence of superior tree performance, particularly because CRF2 reduced fruit yield in 2026. Similar caution has been emphasized in citrus fertilization studies, where nutrient-use efficiency and yield responses are shaped by nutrient rate, nutrient balance, and water-management conditions rather than by fertilizer input alone (Quaggio et al., 2019).

Ridge regression complemented the HCA and PCA by indicating that nutrient–response associations were fertilizer-specific (**Figures 7**, **8**). Rather than identifying a single nutrient consistently associated with yield or juice quality across all treatments, ridge regression showed that the nutritional background underlying each response depended on the fertilization program. This finding supports the use of multi-nutrient diagnostic approaches, which may identify productivity-related nutritional constraints in citrus more effectively than single-nutrient interpretations (Krug et al., 2023). Therefore, ridge regression results should be interpreted as part of an integrated nutritional profile rather than as isolated nutrient effects.

For fruit yield, the positive association between leaf P concentration and yield under CRF1 indicates that, within this fertilization program, higher leaf P status was associated with greater fruit production after accounting for the other macronutrients included in the model (**Figure 7A**). This result indicates that P was among the nutrients most strongly associated with yield variation under CRF1. However, this statistical assocaiteion does not demonstrate that P availability or internal P balance caused the observed variation in fruit yield. The association is nevertheless physiologically plausible, as P plays essential roles in energy transfer, nucleic acid metabolism, root activity, carbohydrate transport, flowering, and fruit development (Obreza et al., 2008; Duan et al., 2022; Khan et al., 2023). In citrus, P nutrition has also been associated with changes in soluble sugar and citric acid metabolism, suggesting that P may influence both productivity and fruit quality-related processes (Wu et al., 2021a).

Under CRF2, fruit yield was positively associated mainly with leaf N and S concentrations (**Figure 7A**). These associations suggest that N and S were more closely linked to yield variation under CRF2, particularly because this treatment showed high apparent PFP-K_2_O but reduced yield in 2026. Nitrogen is directly involved in vegetative growth, photosynthetic capacity, protein synthesis, and yield formation in citrus, and N deficiency has been shown to reduce citrus growth, mineral nutrient status, photosynthesis, and related physiological parameters (Huang et al., 2021). Sulfur also contributes to amino acid and protein synthesis, chlorophyll formation, enzyme activity, and stress-related metabolism (Narayan et al., 2023). Therefore, the positive N–yield and S–yield associations under CRF2 suggest that yield variation was linked to nutrients involved in photosynthesis, protein metabolism, and stress acclimation rather than to K_2_O productivity alone.

The negative association between leaf Ca and yield under CRF2 should be interpreted cautiously (**Figure 7A**). This coefficient does not indicate that Ca directly reduced fruit production or that Ca was excessive. Instead, it indicates that, within CRF2, higher leaf Ca concentrations were associated with lower fruit yield after accounting for the other macronutrients in the model. This pattern may reflect the specific nutritional context of CRF2, where reduced yield, altered leaf nutrient balance, and HLB-related physiological constraints may have modified the relationship between nutrient concentration and fruit production. Higher leaf Ca may also have occurred in trees with lower fruit load, resulting in a concentration effect rather than a direct negative effect of Ca. Because HLB affects citrus root growth, fine root mass, nutrient uptake capacity, and nutrient translocation, leaf nutrient concentrations should not be interpreted as direct evidence of sufficiency or causal effects in HLB-affected trees (Atta et al., 2020a; Hallman et al., 2022).

For juice quality, ridge regression revealed weaker and less consistent nutrient associations than those observed for fruit yield, supporting the univariate results showing that juice quality attributes were not significantly affected by fertilization program (**Figures 7B–E**). This response suggests that juice content, titratable acidity, °Brix, and the °Brix-to-acid ratio were relatively stable compared with fruit yield and nutrient productivity under the conditions of this study. Because citrus fruit quality is governed by multiple interacting factors, including cultivar, crop load, nutrient status, water availability, canopy condition, and environmental conditions, these findings suggest that juice quality variation was not primarily driven by a single leaf nutrient, but rather reflected the combined influence of multiple physiological and environmental factors.

Micronutrient associations also differed among fertilization programs (**Figure 8**). The positive associations of Zn and Fe with yield under CRF2, and of Cu under CRF1, indicate that, within each fertilization program, higher concentrations of these micronutrients were associated with greater fruit production. These responses suggest that micronutrients contributed differently to the nutritional background underlying yield variation, depending on fertilizer composition and nutrient balance. This distinction is particularly relevant because HLB-affected citrus trees commonly exhibit micronutrient deficiencies or imbalances, especially involving Zn, Mn, and Fe (Kwakye and Kadyampakeni, 2022; Kadyampakeni et al., 2023). Thus, the ridge regression results suggest that fertilizer composition and nutrient balance may have shaped the micronutrient context associated with yield, but the coefficients should be interpreted as exploratory associations rather than direct causal effects.

It is important to note that this study was conducted with mature ‘Valencia’ sweet orange trees grafted on Swingle citrumelo rootstock. This rootstock was historically one of the most widely used in Florida citrus production; however, HLB-associated root decline may limit the capacity of trees on Swingle citrumelo to fully respond to improved fertilization programs. Previous studies have reported substantial feeder root loss in HLB-affected trees grafted on Swingle citrumelo, which may reduce nutrient uptake capacity and delay tree responses to enhanced nutrient supply. Therefore, the responses observed in the present study should be interpreted in the context of rootstock-specific limitations imposed by HLB-related root impairment. It is possible that rootstocks with greater tolerance to HLB-associated root decline, such as US-942, US-812, or UFR-5, may show different responses to similar fertilization programs, particularly if improved root persistence supports greater nutrient acquisition and translocation. In addition, fruit yield responses in 2025 and 2026 may have been influenced by adverse weather events, including Hurricane Milton and seasonal freezing conditions, which likely contributed to the lower overall yields observed during these years. These environmental events affected the experimental area as a whole and therefore were not specific to any fertilization program. However, the significant fertilization program × year interaction for fruit yield indicates that the relative response of the fertilization programs was not constant across years, even under the same year-specific environmental conditions. Thus, while adverse weather likely contributed to the overall temporal decline in yield, the lower relative performance of CRF2 in 2026 cannot be attributed solely of the environmental effect of that year.

## 5 Conclusion

This study demonstrates that controlled-release fertilizer programs are not agronomically interchangeable in mature HLB-affected ‘Valencia’ sweet orange trees grown on sandy soil in Florida. CRF1 was the most effective controlled-release strategy, as it maintained fruit yield comparable to the conventional Standard program while promoting a distinct leaf nutritional profile, particularly through higher Mg and S concentrations. In contrast, CRF2 increased apparent K_2_O partial factor productivity but reduced fruit yield in 2026, indicating that higher nutrient productivity indices do not necessarily represent superior agronomic performance when yield stability is compromised. Juice quality attributes were not significantly affected by fertilization programs, suggesting that treatment effects were expressed mainly through leaf nutrition, yield response, and nutrient productivity rather than fruit internal quality. Overall, these findings highlight that fertilizer formulation, nutrient balance, release pattern, and application strategy are critical determinants of CRF performance under HLB-affected field conditions.

## Data Availability

The raw data supporting the conclusions of this article will be made available by the authors, without undue reservation.

## Supplementary material

Supplemental material 1 Soil phosphorus **(a-c)**, potassium **(d-f)**, calcium **(g-i)**, magnesium **(j-l)** and sulfur **(m-o)** at 0-20 and 20-40 cm depth in soil cultivated with ‘Valencia’ sweet orange as affected by fertilization programs (T) from 2024 to 2026. , * and ns: significant at 5% and 1% of probability and non-significant by F-test. Different lowercase letters indicate significant differences among fertilization treatments within each year, determined by Tukey’s honest significant difference (HSD) test at p ≤ 0.05; the absence of letters indicates no significant treatment effect. Bars represent means ± standard error.

Supplemental material 2 Soil pH **(a-c)**, organic matter content **(d-f)**, and cation exchange capacity **(g-i)** at 0-20 and 20-40 cm depth in soil cultivated with ‘Valencia’ sweet orange as affected by fertilization programs (T) from 2024 to 2026. , *, and ns: significant at 5% and 1% of probability and non-significant by F-test. Different lowercase letters indicate significant differences among fertilization treatments within each year by Tukey’s honest significant difference (HSD) test at p ≤ 0.05; the absence of letters indicates no significant treatment effect. Bars represent means ± standard error.

Supplemental material 3 Height **(a-c)**, canopy diameter **(d-f)**, canopy area **(g-i)**, canopy volume **(j-l)**, ratio (height/diameter: **(m-o)**, and skewness index **(p-r)** of ‘Valencia’ sweet orange as affected by fertilization programs (T) from 2024 to 2026. , *, and ns: significant at 5% and 1% of probability and non-significant by F-test. Different lowercase letters indicate significant differences among fertilization treatments within each year, determined by Tukey’s honest significant difference (HSD) test at p ≤ 0.05; the the absence of letters indicates no significant treatment effect. Bars represent means ± standard error.
