# Supplementary material for: Controlled-release fertilizer programs differentially affect leaf nutrition and nutrient factor productivity in mature HLB-affected ‘Valencia’ sweet orange trees in Florida
Source: Front Plant Sci. 2026 Sep 28;17:1932128. doi: 10.3389/fpls.2026.1932128 (PMC13655167; doi:10.3389/fpls.2026.1932128)
Supplement: Supplementary file 1 [file Table1.docx]

**Sup_4.** Mixed-model analysis of the effects of fertilization program, year, and their interaction on soil properties, leaf nutrient concentrations, tree biometric traits, fruit yield, juice quality, and partial factor productivity.

| **Variable** | **Fertilization program** | **Year** | **Fertilization program × Year** |
| --- | --- | --- | --- |
| **Soil properties – 0–20 cm** |  |  |  |
| pH | 0.685 | <0.001 | 0.008 |
| Organic matter | 0.060 | 0.088 | 0.001 |
| CEC | 0.084 | 0.008 | <0.001 |
| P | 0.640 | 0.388 | 0.189 |
| K | 0.962 | 0.005 | 0.515 |
| Ca | 0.024 | 0.197 | <0.001 |
| Mg | 0.840 | 0.049 | <0.001 |
| S | 0.011 | <0.001 | 0.119 |
| **Soil properties – 20–40** **cm** |  |  |  |
| pH | 0.332 | <0.001 | 0.604 |
| Organic matter | 0.157 | 0.359 | 0.148 |
| CEC | 0.165 | 0.406 | 0.453 |
| P | 0.750 | 0.043 | 0.044 |
| K | 0.826 | 0.005 | 0.349 |
| Ca | 0.202 | <0.001 | 0.741 |
| Mg | 0.826 | <0.001 | 0.125 |
| S | 0.436 | <0.001 | 0.399 |
| **Leaf macronutrients** |  |  |  |
| N | 0.067 | <0.001 | 0.356 |
| P | <0.001 | <0.001 | <0.001 |
| K | 0.146 | 0.045 | 0.364 |
| Ca | 0.254 | <0.001 | 0.043 |
| Mg | 0.001 | <0.001 | 0.076 |
| S | 0.154 | <0.001 | 0.027 |
| **Leaf micronutrients** |  |  |  |
| B | 0.978 | <0.001 | 0.041 |
| Zn | 0.661 | <0.001 | 0.516 |
| Mn | 0.908 | <0.001 | 0.121 |
| Fe | 0.791 | 0.165 | 0.964 |
| Cu | 0.562 | <0.001 | <0.001 |
| **Tree biometric variables** |  |  |  |
| Tree height | 0.483 | 0.735 | 0.458 |
| Canopy diameter | 0.755 | 0.005 | 0.185 |
| Canopy area | 0.717 | 0.009 | 0.216 |
| Canopy volume | 0.790 | 0.212 | 0.669 |
| Height-to-diameter ratio | 0.133 | 0.904 | 0.256 |
| Skewness index | 0.038 | <0.001 | 0.171 |
| Fruit yield and juice quality |  |  |  |
| Fruit yield | 0.014 | <0.001 | 0.004 |
| Juice content | 0.380 | <0.001 | 0.534 |
| Titratable acidity | 0.293 | <0.001 | 0.028 |
| Soluble solids (°Brix) | 0.612 | <0.001 | 0.558 |
| °Brix-to-acid ratio | 0.470 | <0.001 | 0.095 |
| **Partial factor productivity** |  |  |  |
| PFP-N | <0.001 | <0.001 | <0.001 |
| PFP-P₂O₅ | <0.001 | <0.001 | <0.001 |
| PFP-K₂O | <0.001 | <0.001 | <0.001 |
